# Copper-Substituted Polyoxotungstates as Catalysts for the Electrocatalytic Oxygenation of Light Alkanes

**DOI:** 10.1021/acs.inorgchem.5c02816

**Published:** 2025-09-04

**Authors:** Yehonatan Kaufman, Ronny Neumann

**Affiliations:** Department of Molecular Chemistry and Materials Science, 34976 Weizmann Institute of Science, Rehovot 7610001, Israel

## Abstract

The low-temperature oxidation of alkanes and arenes using molecular oxygen under ambient conditions is still one of the grand challenges of catalysis. Inspired by the alkane hydroxylation activity of the copper-based metalloenzyme, particulate methane monooxygenase, a tetra-copper polyoxometalate, [Cu_4_(H_2_O)_2_(PW_9_O_34_)_2_]^10–^, was investigated as an electrocatalyst for the cathodic (reductive) oxidation of hydrocarbons with emphasis on oxidation of ethane. Controlled potential electrolysis (CPE) in water at −0.45 V versus NHE showed the formation of ethanol, acetaldehyde, and then acetic acid. Divided cell CPE confirmed that oxidation of ethane to ethanol occurs only at the cathode, while further oxidation can occur at the cathode and anode. Experiments using a chemical reductant ^18^O_2_ and halides confirmed the formation of an active species at the cathode. Different combinations of redox-active copper and redox-inactive zinc within the polyoxometalate framework demonstrate that the O_2_ activation probably requires three copper atoms. The use of kinetic isotope effects and probe molecules indicates a rebound mechanism for aliphatic substrates and an electrophilic species for oxidation of arenes. The transformation of ethane to either ethanol or acetic acid is an important transformation, especially in scenarios where ethane is not recovered from natural gas.

## Graphical abstract

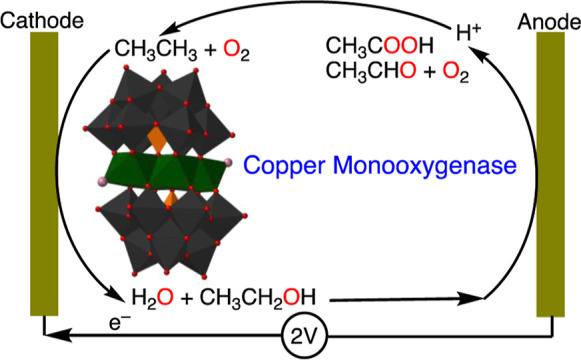

## Introduction

The oxygenation of hydrocarbons with dioxygen from air, especially low-molecular-weight gaseous alkanes found in natural gas, is a major challenge in oxidation catalysis. It also has major implications for improved utilization of natural gas resources that are often flared off with deleterious net emission of carbon dioxide. Among the desirable transformations are alkane oxidation to alcohols, aldehydes, and carboxylic acids.

Methane monooxygenases are bacterial enzymes that can catalyze the aerobic oxidation of alkanes under reducing conditions. There are two main variants of methane monooxygenase: soluble methane monooxygenase (sMMO), which is found in the cytosol and has two iron atoms at the active site, and particulate methane monooxygenase (pMMO), a membrane-bound enzyme with a copper-based active site. The structure of the pMMO active site, as well as the number and position of its copper atoms, is still a matter of considerable discussion and research.^1–8^

The ubiquitous nature of copper centers in monooxygenase enzymes has led to long-standing efforts to find practical, especially inorganic, synthetic analogues for pMMO. One very active research area is the incorporation of copper into zeolites, clays, and metal organic framework materials that has led to the demonstrated carbon–hydrogen bond activation of methane to form methanol via proposed active metal-oxo species. Such use of oxygen from air as an oxidant typically requires a three-step reaction sequence. The zeolite is activated at high temperature under an atmosphere of O_2_; then, it is reacted with methane, and finally, it is treated with steam at elevated temperatures to remove methanol from the catalysts.^9–13^ Based on copper as the catalytic center, the above-mentioned heterogeneous catalysts do not activate O_2_ under reducing conditions but rather by the application of high temperatures.

Dioxygen activation at ambient conditions toward formation of reactive intermediates, followed by actual catalytic transformations, especially of light hydrocarbons and alkanes using intrinsically stable inorganic copper-based catalysts, has only rarely been attained, despite much biomimetic research on dioxygen activation and identification of reaction modes and intermediate species.^14–19^ The notable example, reported by Chan and co-workers, is catalysis by a tricopper organic complex with the use of H_2_O_2_ as a sacrificial reductant^20–22^ and a cathodic electrochemical oxidation with O_2_.^23^

Polyoxometalates have been used in very high temperature oxygenation of alkanes, although selectivity and yields are low with significant formation of combustion products.^24^ Recently, we have shown that iron-tungsten Keplerates, a subclass of polyoxometalate compounds, can activate O_2_ and oxygenate light alkanes, e.g., ethane to acetic acid, arenes, alkenes, ketones, sulfides, and amines, in a cathodic electrochemical transformation where a compound I-type intermediate has been suggested as the reactive species.^25,26^ In this context, such electrochemical reducing conditions can, in principle, allow an on-site, at the natural gas well, oxidation of natural gas components in relatively small reaction units that require only water, air, and electricity, where the latter could also be of a renewable nature. Thus, the main objective of this research was to study the cathodic electrocatalytic hydroxylation of light alkanes, with emphasis on ethane, using O_2_ as an oxidant. Here, we have prepared a series of copper-containing polyoxometalates that are electrocatalysts for the oxidation of light alkanes; Figure 1. The most active and most studied compound was the Weakley-type tetra-copper-substituted anions, [Cu_4_(H_2_O)_2_(PW_9_O_34_)_2_]^10–^, or {Cu_4_(PW_9_)_2_} in shorthand notation; Figure 1.^27,28^ The {Cu_4_(PW_9_)_2_} polyoxometalate is composed of a ring of four copper atoms “sandwiched” between two phosphotungstate moieties.

**Figure 1:**
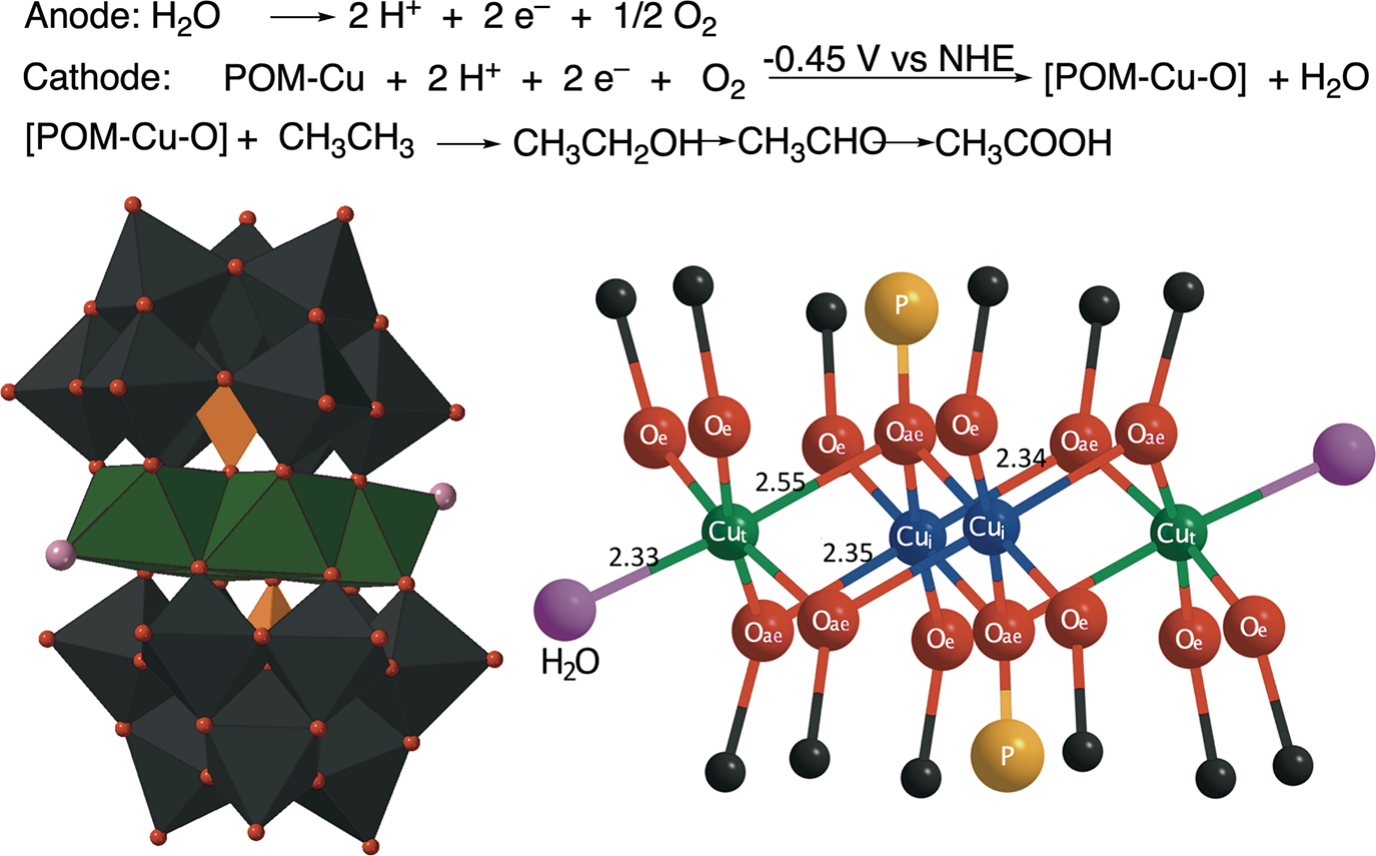
Top—cathodic activation of dioxygen and hydroxylation of ethane catalyzed by the tetra-copper-substituted polyoxometalate, {Cu_4_(PW_9_)_2_}. Bottom—a polyhedral representation of the catalyst (left) and ball-and-stick representation of its copper “ring” (right). P—orange; W—dark gray; Cu—green/blue; O—red; H_2_O—pink.

## Results and Discussion

The initial reactivity of O_2_ toward {Cu_4_(PW_9_)_2_} under reducing conditions was evaluated by cyclic voltammetry (CV); Figure 2. As can be seen in Figure 2A, in the absence of polyoxometalate, the one-electron reduction peak of molecular oxygen to superoxide O_2_ → O_2_^•–^ appears at −0.52 V vs Ag/AgCl. The analogous tetra-zinc compound of {Zn_4_(PW_9_)_2_} shows a small reversible peak at about −0.9 V attributable to a W^VI^ ↔ W^V^ redox couple when scanned under an atmosphere of argon. In the presence of 1 bar of molecular oxygen, the O_2_ → O_2_^•–^ reduction peak is observed at −0.68 V, somewhat shifted to a more negative potential compared to the O_2_ → O_2_^•–^ reduction peak in the absence of the tetra-zinc polyoxometalate; Figure 2B.

**Figure 2:**
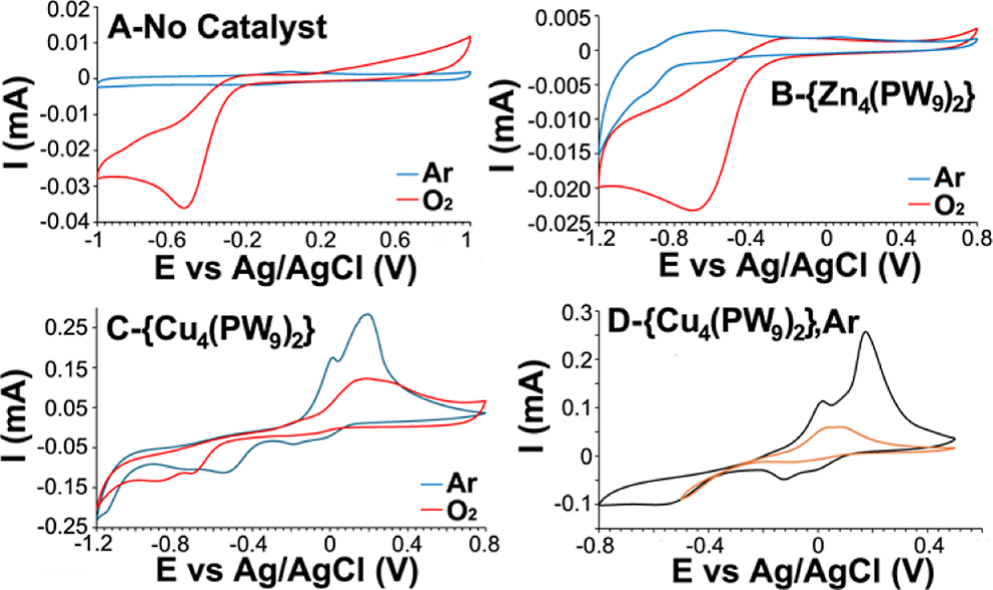
CV of {Cu_4_(PW_9_)_2_} and relevant controls in water. Conditions—10 mL of analyte solution containing a 0.1 M supporting electrolyte. Working electrode (WE)—glassy carbon disc, counter electrode (CE)-Pt wire, reference electrode (RE)—Ag/AgCl. (A) Control CV of 0.1 M NaClO_4_ under argon (blue) and O_2_ (red); (B) CV of 4 mM {Zn_4_(PW_9_)_2_} in a 0.1 M NaClO_4_ solution under argon (blue) and O_2_ (red); (C) CV of 4 mM {Cu_4_(PW_9_)_2_} in a 0.1 M NaClO_4_ solution under argon (blue) and O_2_ (red); (D) CV 4 mM {Cu_4_(PW_9_)_2_} in 0.1 M NaClO_4_ under argon at two different potential windows.

The CV of {Cu_4_(PW_9_)_2_}, Figure 2C, under 1 bar of Ar shows multiple redox transitions. First, there are two quasi-reversible peaks associated with a Cu^2+^ ↔ Cu^1+^ transition at −0.03 V and at −0.1 8 V. The two peaks are so assigned because {Cu_4_(PW_9_)_2_} contains two distinct copper sites—two copper atoms are at the internal position and two are at terminal positions (see Figure 1 where the internal and terminal copper atoms are labeled and color-coded blue and green, respectively; see below for more clarification regarding the copper sites). Under 1 bar of Ar, at more negative potentials of −0.6 V to −0.8 V are peaks that one can assign to the Cu^1+^ → Cu^0^ reduction. The assignment of those peaks is supported by the strong reoxidation peak at 0.2 V associated with what is often referred to as anodic Cu “stripping”—an oxidation of electrode-adsorbed Cu^0^ species. This “stripping wave” is not observed when carrying out the CV in a smaller potential window where there is no reduction to Cu^0^; Figures 2D and S4. The CV of {Cu_4_(PW_9_)_2_} under O_2_ changes dramatically; Figure 2C. Notably, the Cu^2+^ ↔ Cu^1+^ transition is no longer observable, indicating a reaction between Cu^1+^ and O_2_, e.g., formation of Cu^II^O_2_^•–^, that one may assume is the first step in a presumptive catalytic cycle. As will be shown below, the reduction of Cu^II^O_2_^•–^ is the potential at which a maximum catalytic oxygenation takes place; Figure S2. Also, there is the observation that there is no apparent anodic Cu^0^ “stripping” under O_2_ but possibly a reoxidation peak of a [Cu_*x*_–O_2_] species. There are two additional irreversible reduction peaks at 1 bar O_2_ at more negative potentials that one can assign to a W^VI^ ↔ W^V^ redox couple. This assignment is supported by the peaks observed for {Zn_4_(PW_9_)_2_} and the very similar potential of the W^VI^ ↔ W^V^ redox couple reported for [Cu_4_(H_2_O)_2_(P_2_W_15_O_56_)_2_]^16–^ in water.^29^

The reactivity of several copper-containing polyoxometalates was surveyed by carrying out controlled potential electrolysis (CPE) experiments using ethane as the substrate in water in an undivided cell configuration. As can be seen from the results in Table 1, {Cu_4_(PW_9_)_2_} yielded the most product with a high selectivity of 84% to acetic acid and a moderate faradaic efficiency. The catalyst was stable under reaction conditions and could be reused; see Supporting Information. Reactions at different potentials showed a maximum at −0.45 V versus NHE (a cell voltage of 2 V); Table S1. An analogous, more extended sandwich-polyoxometalate [Cu_4_(H_2_O)_2_(P_2_W_15_O_56_)_2_]^16–^ ^28^ was significantly less reactive despite having an identical tetra-copper ring. Its low reactivity can be attributed to its bulkiness and larger negative charge, which might hinder the cathodic reduction, emphasizing the role of the polyoxotungstate framework (and not just the copper ring) in the reaction.

**Table 1:** Cathodic Oxidation of Ethane Catalyzed by Various Copper-Containing Polyoxometalates

	product, μmol			
catalyst	CH_3_CH_2_OH	CH_3_CHO	CH_3_COOH	F.E. %
{Cu_4_(PW_9_)_2_}	1.27	2.96	19.6	45
{Cu_4_(P_2_W_15_)_2_}	0.13	0.09	0.52	0.72
{Cu_3_(PW_9_)_2_}	0.50	2.1	11.3	43
{Cu_3_(BiW_9_)_2_}^a^	0.9	n.d.	2.3	2.8
{Cu_3_SiW_9_}	0.42	0.26	1.9	4.4
{NaF_6_CuW_17_}	trace	trace	0.1	<1
{Cu_2_PW_10_}	n.d.	n.d.	n.d.	n.d.

a Reaction conditions: catalysts (10 μmol) were dissolved in 2.5 mL of D_2_O inside an airtight electrochemical cell. A three-electrode configuration was used. Working electrode (cathode)-Pt net, a surface area of about 20 cm^2^ (calculated using chemisorption of H^+^ to the electrode; Figure S3); counter electrode (anode)—Pt wire, RE—Pt wire; 1 bar of air; 2 bar of ethane; cathodic potential −0.45 V vs NHE; ∼22 °C; 24 h. There is no reaction in the absence of a copper-based polyoxometalate. Liquid phase product analysis by ^1^H NMR using dimethyl sulfone as the internal standard introduced postreaction. Gas phase analysis using gas chromatography with a thermal conductivity detector (GC-TCD) showed no formation of CO or CO_2_. n.d.—not detected. (a) A two-electrode configuration was used with a cell potential of −2.3 V—corresponding to a cathodic potential of −0.75 V vs NHE.

The tricopper Knoth-type anion, [Cu_3_(PW_9_O_33_)_2_]^12–^, was also quite reactive although apparently less stable,^30^ but the iso-structural [Cu_3_(H_2_O)_3_(BiW_9_O_33_)_2_]^12–^ was much less reactive.^31^ Experimentally, it was found that the bismuth analogue requires higher overpotential to operate, indicating that the heteroatom inside the polyoxometalate moiety and the concomitant Bi­(III) electron lone pair influence its reduction potential. The dicopper-substituted Keggin-type [Cu_2_(H_2_O)_2_PW_10_O_38_]^8–^, was inactive.^32^ The quasi-Wells–Dawson fluorinated polyoxometalate, [NaF_6_Cu­(H_2_O)­W_17_O_55_]^15–^, that can stabilize high valent oxidation states showed low reactivity.^33,34^ The tricopper-substituted polyoxometalate of [SiCu_3_(H_2_O)_3_W_9_O_37_]^8–^ was initially reactive, as also indicated by its CV, Figure S5,^35^ but tended to be unstable under reducing conditions in water. The improved stability of {Cu_4_(PW_9_)_2_} vs [SiCu_3_(H_2_O)_3_W_9_O_37_]^8–^ indicates the importance of the double-sided bond between the copper atoms and the polyoxometalate moieties in the former. See Figure S6 for the molecular structures of the various compounds. From these initial survey studies, it would appear that at least two, but more likely three copper atoms, are involved in the electrocatalytic oxygenation reaction, although other factors may be at play. Further CPE experiments were carried out on the oxidation of methane using the preferred {Cu_4_(PW_9_)_2_} as a catalyst; Scheme 1. Using a Parr autoclave fitted with electrodes, one can observe that at 2 bar of methane, there is hardly any product, but an increase of pressure and, thereby, an increase in the methane solubility in water, significantly increases both the product yield of both methanol and formic acid, and the Faradaic efficiency. Further oxidation reactions of propane, ethylene, and propylene are presented in the Supporting Information, Schemes S1–S3.

**Scheme 1:**
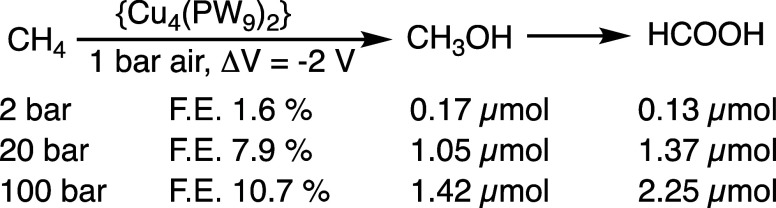
Cathodic Oxidation of Methane at Various Pressures Catalyzed {Cu_4_(PW_9_)_2_}­^a^

a Reaction conditions: {Cu_4_(PW_9_)_2_} (10 μmol) was dissolved in 2.5 mL of D_2_O in a Parr autoclave. A 2-electrode configuration was used. WE (cathode)—Pt net, surface area 20 cm^2^; counter electrode (anode)—Pt wire; 1 bar of air; cell potential 2 V; ∼22 °C; 24 h. Liquid phase product analysis by ^1^H NMR using dimethyl sulfone as the internal standard introduced postreaction. Gas phase analysis using gas chromatography with a thermal conductivity detector (GC-TCD) showed no formation of CO or CO_2_.

The oxidation of ethane was postulated (Figure 1 and below) to occur through the formation of an active copper-centered oxygen donor at the cathode, as supported by the CV measurements. However, in an undivided cell configuration, it is conceivable that anodic oxidation of ethane could also occur, for example, by the reaction of ethane with a hydroxyl radical intermediate formed during the water cleavage reaction. More likely, it is possible that after the first 2-electron oxidation of ethane to ethanol, the latter and its subsequent 4-electron oxidation product, acetaldehyde, could also be oxidized at the anode. Thermodynamic calculations at pH 7 show that the anodic potentials for oxidation of ethanol and acetaldehyde are significantly lower than those for the oxidation of water, eqs 1–3

1 $$H_{2}O\to 2H^{+}+2e^{-}+1/2O_{2},E_{0}=0.816\;V$$

2 $${CH}_{3}{CH}_{2}OH\to {CH}_{3}CHO+2H^{+}+2e^{-},E_{0}=0.02\;V$$

3 $${CH}_{3}CHO+H_{2}O\to {CH}_{3}COOH2H^{+}+2e^{-},E_{0}=0.56\;V$$

To probe the possibility of an anodic oxidation reaction, a divided cell configuration is required. Typical H-cell configurations using membranes such as Nafion are not suitable because of the relatively fast diffusion of ethanol, acetaldehyde, and acetic acid between the cathode and the anode cell compartments. An improvement could be obtained, albeit at much lower currents and product yields, by using an agarose salt bridge to separate the cathode from the anode; Figure S1. The diffusion rate of small organic molecules through the bridge was significantly slower but still measurable. In the typical reaction time scale of 18 h, it was separately observed that mostly ethanol was able to diffuse from one compartment to the other, but acetaldehyde and acetic acid only diffused in very small amounts.

Cathodic versus anodic oxidation reactions were probed by using various combinations of catalytically active {Cu_4_(PW_9_)_2_} and isoelectronic and isostructural but catalytically inactive {Zn_4_(PW_9_)_2_}. The results are summarized in Table 2. The following conclusions can be made: (i) ethane is only oxidized at the cathode, as no oxidation products were detected when catalyst {Cu_4_(PW_9_)_2_} was presented only in the anodic compartment. As expected, {Zn_4_(PW_9_)_2_} did not produce any products in the cathode compartment. (ii) Ethanol can be oxidized both at the anode and at the cathode. As we stated above, ethanol can diffuse through the salt bridge but acetaldehyde and acetic acid cannot, so the presence of acetaldehyde in both anodic and cathodic compartments indicates that it formed there. Ethanol oxidation to acetaldehyde is more efficient at the anode since no ethanol was detected in the anolyte, but ethanol was detected in the catholyte. This is supported by the significantly lower potential required for anodic oxidation of ethanol versus water. (iii) Oxidation of acetaldehyde to acetic acid occurs at both the cathode and anode. (iv) The anodic oxidation of ethanol and acetaldehyde is catalyzed at the Pt anode but not significantly by the polyoxometalate since both {Zn_4_(PW_9_)_2_} and {Cu_4_(PW_9_)_2_} gave similar results.

**Table 2:** Oxidation of Ethane in a Divided Cell with an Agarose Bridge^a^

	compound	EtOH, μmol	MeCHO, μmol	AcOH, μmol
cathode	{Zn_4_(PW_9_)_2_}	n.d.	n.d.	n.d.
anode	{Cu_4_(PW_9_)_2_}	n.d.	n.d.	n.d.
cathode	{Cu_4_(PW_9_)_2_}	0.44	0.84	0.19
anode	{Zn_4_(PW_9_)_2_}	n.d.	0.21	0.16
cathode	{Cu_4_(PW_9_)_2_}	0.50	0.93	0.26
anode	{Cu_4_(PW_9_)_2_}	n.d.	0.24	0.15

a Reaction conditions: 4 mM {Cu_4_(PW_9_)_2_} or {Zn_4_(PW_9_)_2_} was dissolved in 3 mL of D_2_O and placed in the relevant compartment. The entire cell was pressurized with 2 bar of ethane and 1 bar of air at room temperature. The cathode was a platinum net, and the anode was a platinum wire; cell potential 2 V, *t*—18 h. n.d.—not detected.

Several additional lines of evidence for the reductive activation of O_2_ to form a copper-based active species are presented. First, ethane oxygenation can be obtained by using a reducing agent, sodium thionate, instead of using an electrochemical setup. Thus, in a glass Fisher–Porter pressure tube, {Cu_4_(PW_9_)_2_} (10 μmol) and NaS_2_O_4_ (30 μmol) were dissolved in 2.5 mL of D_2_O. The pressure tube was pressurized with 1 bar of air and 4 bar of ethane. After 6 h at room temperature, analysis of the reaction mixture by ^1^H NMR showed the formation of 0.19 μmol of ethanol and 0.04 μmol of acetaldehyde. Second, a CPE experiment using 98% 18-O-labeled ^18^O_2_ for the oxidation of phenol was carried out. Thus, {Cu_4_(PW_9_)_2_} (10 μmol) was dissolved in 2.5 mL of D_2_O and 80 μmol of phenol was added. The cell was degassed and filled with 2 atm of ^18^O_2_. Note that phenol was used as a substrate since oxygen atoms of acetic acid (formed by ethane oxidation) are easily exchanged by reaction with water. Using a platinum net cathode and a Pt wire anode at a 2 V cell potential for 18 h, the CPE yielded 15.9 μmol of catechol and 16.1 μmol of hydroquinone as quantified by ^1^H NMR. The ratio between ^16^O and ^18^O containing products was determined using GC–MS and showed a 78% ^18^O incorporation into the products. The dilution of the isotope incorporation could be due to the formation of ^16^O_2_ from the anodic oxidation of H_2_^16^O and/or fast exchange of an ^18^O atom of the active species with H_2_^16^O.^26^

Finally, we observed that {Cu_4_(PW_9_)_2_} can activate halides under aerobic conditions and promote the halogenation of aromatic substrates. Using a divided cell with an agarose salt bridge, NaCl along with phenol was placed in the cathode cell only. A CPE catalyzed by {Cu_4_(PW_9_)_2_} showed the formation of chlorinated phenols as shown in Scheme 2. Since there have been reports of aerobic oxychlorination of arenes with LiCl, O_2,_ and Cu^II^ salts,^36–38^ albeit at higher temperatures in acetic acid, a control experiment with 2 bar of O_2_ under open circuit conditions at room temperature for 20 h was carried out and showed no formation of halogenated phenol. This supports the conclusion that an active copper oxygen species formed at the cathode leads to halide oxidation and in situ oxyhalogenation of phenol. The preference toward multiple halogenations of the substrate (especially when using bromide) indicates the formation of electrophilic halogen species.

**Scheme 2:**
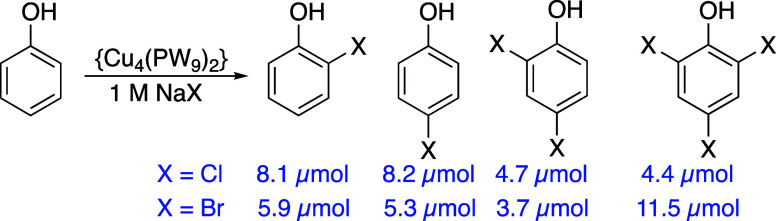
Cathodic Oxyhalogenation of Phenol in a Divided Cell with an Agarose Bridge^a^

a Reaction conditions: 4 mM {Cu_4_(PW_9_)_2_}, 0.25 M phenol, and 1 M NaX were dissolved in 3 mL of H_2_O and placed in the cathode compartment. CPE was carried out under ambient air using a Pt net cathode and a Pt wire anode at a cell potential of 2 V for 20 h. The products were identified by GC–MS and quantified by GC-TCD.

Another topic of interest relates to the number of copper atoms involved in the catalytic reaction. From the X-ray structure for {Cu^II^_4_(PW_9_)_2_} shown in Figure 1,^28^ there are two different types of Cu atoms, those in terminal positions, Cu_t_, with a labile aqua ligand in the axial plane and those that are in internal positions, Cu_i_, the access to which are sterically hindered. In the somewhat bent equatorial plane, the Cu–O_e_ distances are in the range of 1.9–2.0 Å. However, notable is the fact that the Cu–O atom distances in the axial planes Cu–O_ae_ and Cu–OH_2_ are both long, and therefore, the coordination around the Cu atoms could be considered to be nearly square planar; see Figure 1. To assess how many copper atoms are involved in the reaction, we decided to use mixed-ring analogues of {Cu_4_(PW_9_)_2_}, where some of the copper atoms will be substituted by inactive zinc atoms. First, a mixed metal Zn–Cu polyoxometalate, {Cu_*x*_Zn_4–*x*_(PW_9_)_2_} *x* = 0–4, was prepared by using equimolar amounts of Zn^II^ and Cu^II^. Assuming that each position is occupied randomly by either Cu or Zn atoms, one can expect nine different products, as shown in Figure 3. Parallel separate reactions were carried out with {Cu_4_(PW_9_)_2_} and {Cu_*x*_Zn_4–*x*_(PW_9_)_2_} as the catalysts. As shown in Scheme 3, {Cu_*x*_Zn_4–*x*_(PW_9_)_2_} showed only about ∼18% of the activity of {Cu_4_(PW_9_)_2_}. A first approximation useful to explain the results can be attained from the distribution of compounds in {Cu_*x*_Zn_4–*x*_(PW_9_)_2_}. Reasonably, one can expect that O_2_ is most likely to bind at a terminal Cu atom by ligand exchange with the aqua ligand. Then, further reaction may reasonably be attributed to interactions including electron transfer with the nearest neighbor Cu atoms at the internal positions. Thus, species {Cu_4_(PW_9_)_2_} (6.25%) and {Cu_t_Cu_i2_(PW_9_)_2_} (12.5%) together could account for the activity of “{Cu_*x*_Zn_4–*x*_(PW_9_)_2_}” versus {Cu_4_(PW_9_)_2_}. It is important to note that the exact composition of the {Cu_*x*_Zn_4–*x*_(PW_9_)_2_} mixture cannot be measured directly. However, evidence of the presence of multiple species in the mixture can be obtained using ^31^P NMR; see Supporting Information and Figure S7.

**Figure 3:**
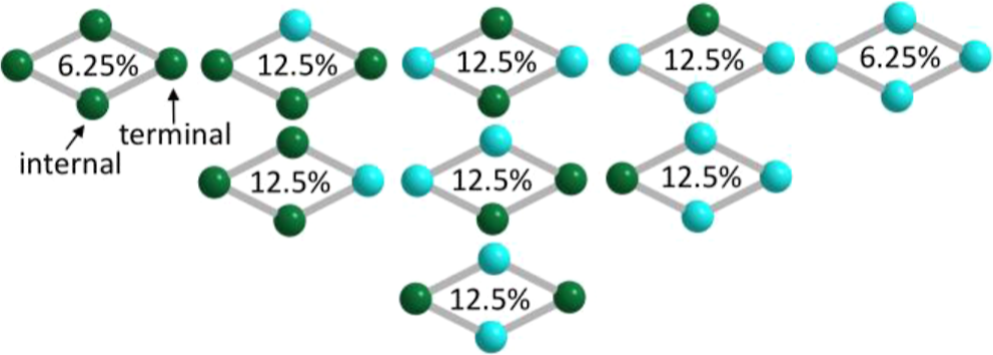
Distribution of positional isomers of {Cu_*x*_Zn_4–*x*_(PW_9_)_2_. Turquoise-Zn; green-Cu. The distribution of compounds is based on the hypothesis that each position is occupied randomly by either Cu or Zn.

**Scheme 3:**
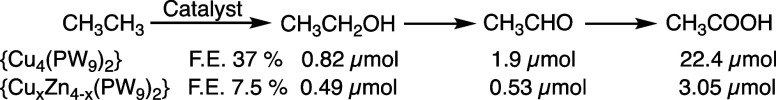
Comparative Activity of {Cu_4_(PW_9_)_2_} and {Cu_*x*_Zn_4–*x*_(PW_9_)_2_} in the Oxidation of Ethane^a^

a Reaction conditions: catalysts (10 μmol) were dissolved in 2.5 mL of D_2_O inside an airtight 3-electrode electrochemical cell. WE (cathode)—Pt net, surface area 20 cm^2^; CE (anode)—Pt wire, RE—Pt wire; 1 bar of air; 2 bar of ethane; cathodic potential −0.45 V; ∼22 °C; 24 h. Liquid phase product analysis by ^1^H NMR using dimethyl sulfone as the internal standard introduced postreaction.

To improve our understanding of the role of each copper atom, a synthetic route that will give us a single product with a well-defined structure rather than having an ensemble of products with different compositions was needed. There are various published examples of mixed-metal phosphotungstate polyoxometalates, most of which are composed of two trilacunary Wells–Dawson moieties of (P_2_W_15_O_56_), that have the general structure of {M″_2_M′_2_(P_2_W_15_O_56_)_2_}, where M″ is the metal at the terminal position and M′ is in the internal bridging position (see Figure S3c). Those polyoxometalates were typically synthesized in two steps. First, a precursor polyoxometalate was synthesized, with the metal M′ at the bridging position and labile sodium or lithium ions at the terminal positions. In the consecutive step, the labile ions are replaced by the desired metal M″ at the terminal position. Various M′M″ combinations for polyoxometalates are known.^39–42^ Mixed-metal “sandwich”-type Keggin phosphotungstates, {M″_2_M′_2_(PW_9_)_2_}, have been less extensively reported in the literature, but they are suitable analogues to our copper catalyst. Compounds with two terminal alkali metal ions (either sodium or lithium) and either zinc, cobalt, nickel, or manganese at the internal positions have been reported.^43^

First, the sodium–zinc polyoxometalate of {Na_2_Zn_2_(PW_9_)_2_} was prepared by the published method, and the {Na_2_Cu_2_(PW_9_)_2_} polyoxometalate was synthesized by adapting the procedure with a copper precursor. Those polyoxometalates have sodium atoms at the terminal positions and either zinc or copper atoms at the internal position. They both were characterized by IR and their CVs; Figures S8–S10. The IR spectrum of {Na_2_Cu_2_(PW_9_)_2_} shared the characteristic peak at 1010 cm^–1^, which is typical for {Cu_4_(PW_9_)_2_}, and its CV was similar to that of {Cu_4_(PW_9_)_2_}. As expected under the conditions noted in Table 1, {Na_2_Zn_2_(PW_9_)_2_} was inactive, while {Na_2_Cu_2_(PW_9_)_2_} showed only minimal activity, 0.11 μmol of ethanol, 0.06 μmol of acetaldehyde, and 0.23 μmol of acetic acid with a low faradaic efficiency of 1.1%. This could be expected as the internal copper atoms are probably quite inaccessible to the O_2_ coordination.

A more interesting case is the terminal copper-substituted polyoxometalate of {Cu_2_Zn_2_(PW_9_)_2_}, which has copper atoms at the terminal positions and inactive zinc atoms at the internal position. It was prepared by the substitution of sodium with copper in {Na_2_Zn_2_(PW_9_)_2_}.^39^ The IR spectrum of {Cu_2_Zn_2_(PW_9_)_2_}, Figure S9, differs from the spectrum of its precursor, {Na_2_Zn_2_(PW_9_)_2_}, but it is similar to the spectrum of {Cu_4_(PW_9_)_2_} indicating the substitution of sodium by copper. In addition, ICP–OES measurement supports the presence of an ∼1:1 ratio of Cu/Zn in this compound; Table S2. Also, here activity was low, 0.29 μmol of ethanol, ∼0.01 μmol of acetaldehyde, and 0.63 μmol of acetic acid with a low faradaic efficiency of 1.5%. These results here support the conclusion we obtained using the ensemble mixture, “{Cu_*x*_Zn_4–*x*_(PW_9_)_2_}”; both a terminal and at least one, likely two, internal copper atoms are needed for the formation of an O_2_-based active species reactive for hydroxylation.

The formation of a plausible initial species between a reduced catalyst, {Cu_4_(PW_9_)_2_}_red,_ and O_2_ eluded us; however, the formation of such a species was observable by UV–vis spectroscopy by reacting {Cu_4_(PW_9_)_2_} with KO_2_ as a reducing agent and then an O_2_ donor. As shown in Figure 4, by subtracting consecutive spectra from the spectrum of {Cu_4_(PW_9_)_2_} upon addition of KO_2_, an isosbestic point at 670 nm appears, indicating likely formation of an O_2_-containing species. Unfortunately, isolation of the species formed was not realized using spectroscopic techniques such as Raman, IR, and EPR spectroscopy.

**Figure 4:**
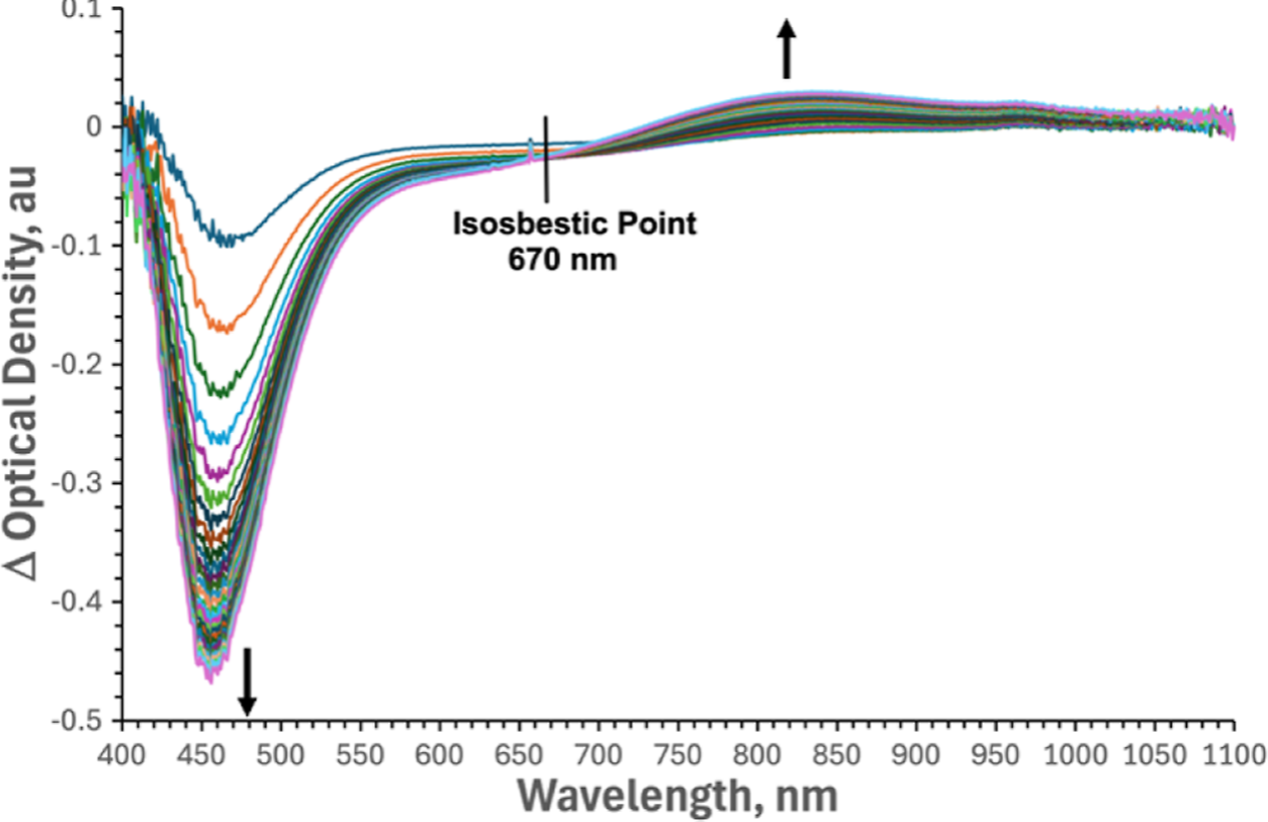
Consecutive visible spectra of 4 mM {Cu^II^_4_(PW_9_)_2_} upon addition of 4 equiv of KO_2_.

In lieu of the identification of the active donor oxygen species formed under reductive conditions, reactivity studies with various kinds of substrates were carried out. First, kinetic isotope effect (KIE) experiments were carried out by reacting a 1:1 mixture of 1,2-ethanediol and 1,2-ethanediol-d4. Thus, 32 μmol of {Cu_4_(PW_9_)_2_} were dissolved in 8 mL solution of 90% H_2_O and 10% D_2_O. 1,2-Ethanediol and 1,2-ethanediol-d4 (10 μmol each) were added. A two-electrode setup was used with a Pt net WE and a Pt wire CE. CPE experiments were performed for 8 h by applying a potential difference of −2 V between the electrodes. Samples of 0.5 mL were extracted every hour, and the consumption of both substrates was analyzed via ^1^H NMR and ^2^H NMR. From the results shown in Figure S11, a KIE of 2.1 was calculated. This result indicates the C–H bond cleavage to be the rate-determining step, and one can posit that the mechanism includes an initial H atom abstraction by the active “Cu–O” species, followed by fast oxygen rebound.

It was surmised that reductive aerobic oxidation of arenes having very strong C–H bonds such as benzene could also be catalyzed by {Cu_4_(PW_9_)_2_}. As shown in Scheme 4, benzene yielded a phenol and further oxygenated products, catechol and hydroquinone, but not further oxidation to quinones by a PCET reaction at the anode. On the other hand, the C–C bond cleavage was observed, as evidenced by the formation of formic acid. In this case, the measurement of a KIE for the oxygenation at a C–H arene bond is also instructive. Because of the limited solubility of benzene, phenol was chosen as the substrate. As described above, a competitive 1:1 mixture of phenol and phenol-d6 was reacted. Thus, under competitive reaction conditions, 32 μmol of {Cu_4_(PW_9_)_2_} was dissolved in 8 mL of D_2_O; 9 μmol of phenol and phenol-d6 each were added. A CPE experiment using a cell potential of 2 V between a Pt net WE and a Pt wire CE was carried out for 9 h, during which 9 samples of 0.25 mL were extracted. The conversion of phenol was analyzed by GC–MS. The results, Figure S12, lead to a calculated KIE = 1.0 consistent with an analogue of an electrophilic aromatic substitution reaction, where the cleavage of the C–H bond is not the rate-determining step. We assume that hydroxylation of a sp^2^ C–H bond occurs via initial formation of a σ bond between the sp^2^ carbon and the active “Cu–O” specie.

**Scheme 4:**
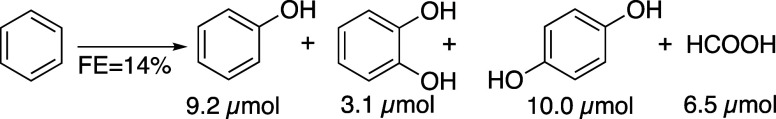
Oxidation of Benzene Catalyzed by {Cu_4_(PW_9_)_2_}­^a^

a Reaction condition: {Cu_4_(PW_9_)_2_} (10 μmol) was dissolved in 2.5 mL of D_2_O inside an airtight electrochemical cell. A 3-electrode configuration was used. WE (cathode)—Pt net, surface area 20 cm^2^; CE (anode)—Pt wire, RE–Pt wire; 1 bar of air; 0.2 mL of benzene; cathodic potential −0.40 V; ∼22 °C; 24 h. Product analysis by ^1^H NMR using dimethyl sulfone as the internal standard introduced postreaction.

Kinetic isotope effect measurements by themselves are not conclusive for a specific active species or mechanism of the reaction. Thus, it is worthwhile noting that for hydroxylation of aliphatic C–H bonds, both high and low KIEs, which can be temperature dependent, have been measured for isolated copper species in organic solution.^44–46^ Somewhat more conclusively, *k*_H_/*k*_D_ = 1 has also been measured for benzene hydroxylation with a viable Cu-based active species.^47^ It is also worthwhile noting that the classic formation of a hydroxyl radical via the Fenton reaction, as originally proposed by Haber and Weiss^48^ shows a KIE *k*_H_/*k*_D_ = 1.7 for benzene hydroxylation.^49^ Another notable comparison is between Fe and Cu tungstate-based polyoxometalates. The iron tungsten polyoxometalate, {Fe_30_W_72_}, shows a KIE = 12.2 for the hydroxylation of 1,2-ethanediol and a KIE = 1 for hydroxylation of phenol.^26^ The difference in the KIE values between Cu and Fe tungstates might arise from the difference in the hydrogen atom acceptor, a difference reflected in the transition state of Cu versus Fe active species. It is interesting that a similar difference was observed for the iron-based sMMO and the copper-based pMMO, where KIE values of 19 and 5.2 for methane/ethane hydroxylation were measured, respectively.^5,50^ Even though our polyoxometalate catalysts are obviously different compared with the enzymes, there are apparently parallel, intrinsic differences between copper and iron active species.^51^

To strengthen the hypothesis of a Cu-based active species versus a hydroxyl radical active species, the reactivity of 1,4-difluorobenzene was studied. The reaction of a Cu-based oxygenating species with arenes proceeds by attack at the ipso position, leading to a keto intermediate via the formation of a σ-complex. Depending on the substituent, substituent migration can occur (NIH shift), but loss of the ipso substituents is typically the major pathway for haloarene hydroxylation, presumably because halides are better leaving groups and weak nucleophiles, especially when solvated in water.^52,53^ In contrast, the Fenton system or others contain an iron salt with peroxide or O_2_/reductant such as Udenfriend, Hamilton, or Viscontini reactions—no ipso attack was observed.^46,54,55^ Indeed, using the reaction conditions described for the oxidation of benzene, the oxidation of 1,4-difluorobenzene yielded only dehalogenated products, 3.6 μmol of 4-fluorophenol and 4.5 μmol of hydroquinone as shown in Scheme 5.

**Scheme 5:**
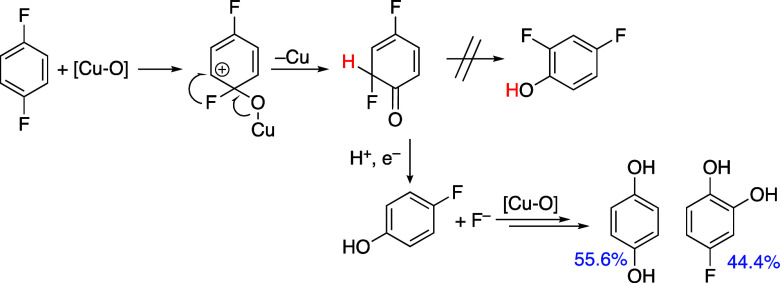
Oxidation of 1,4-Difluorobenzene Catalyzed by {Cu_4_(PW_9_)_2_}­^a^

a Reaction condition: {Cu_4_(PW_9_)_2_} (10 μmol) was dissolved in 2.5 mL of D_2_O inside an airtight electrochemical cell. WE (cathode)—Pt net, surface area 20 cm^2^; CE (anode)—Pt wire, RE-Pt wire; 1 bar of air; 0.5 mL 1,4-difluorobenzene; cathodic potential −0.40 V; ∼22 °C; 24 h. Product analysis by ^1^H NMR using dimethyl sulfone as the internal standard introduced postreaction. [Cu–O] is a general representation of an active species, not a specific species.

The stereochemical outcome of the oxygenation of a *cis*-alkene that can yield either *cis*- or *trans*-epoxide can shed light on the mechanism of the oxygen transfer reaction. Conservation of a *cis*-geometry argues for a fast oxygen atom transfer before formation of a planar intermediate that would lead to the formation of the more thermodynamically stable *trans*-product.^56–58^

The degree of stereo retention in an epoxidation reaction was probed by using a water-soluble *cis*-stilbene to observe the initial epoxide formed before further C–C bond cleavage of the intermediate bond cleavage as observed for ethylene and propylene, Schemes S2 and S3. The electrochemical oxidation of 4,4′-*cis*-stilbene dicarboxylate sodium salt as analyzed after 2 h by ^1^H NMR showed a peak associated with 20 μmol of *cis*-epoxide at 4.24 ppm but no peak at less than 4.0 ppm that could be attributed to the *trans*-epoxide,^59^ in addition to the formation of 100 μmol of C–C bond cleavage products; Scheme 6. The conservation of the *cis*-geometry supports a fast oxygen atom transfer reaction between an active Cu-based species and an alkene and possibly a concerted reaction mechanism.

**Scheme 6:**
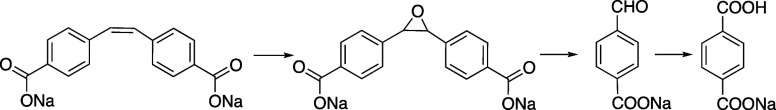
Oxidation of 4,4′-*Cis*-stilbene Dicarboxylate Sodium Salt Catalyzed by {Cu_4_(PW_9_)_2_}­^a^

a Reaction condition: {Cu_4_(PW_9_)_2_} (10 μmol) was dissolved in 3 mL of D_2_O inside an airtight electrochemical cell. WE (cathode)—Pt net, surface area 2.5 cm^2^; CE (anode)—Pt wire, RE—Pt wire; 1 bar of air; 0.075 μmol (25 mM) of 4,4′-*cis*-stilbene dicarboxylate sodium salt; cathodic potential −0.40 V; ∼22 °C; 2 h.

Typically, oxidation of thioethers can result in either S-oxidation or S-dealkylation and this has also been observed for anodic electrochemical reactions.^60^ A strong oxygen donor will oxygenate the sulfur atom, resulting in the corresponding sulfoxide and then sulfone. On the other hand, if the oxidant is either a strong electron acceptor or hydrogen atom acceptor, it will tend to oxidize the alkyl groups at the α position via a proton coupled electron transfer, a hydrogen atom transfer, or a sequential electron transfer proton transfer resulting in the formation of an alpha hydroxylated group, which will eventually decompose to yield a dealkylated product.^61^ In order to probe the properties of the active species formed from {Cu_4_(PW_9_)_2_} at the cathode, the oxidation of thioanisole was carried out in a divided cell configuration, Scheme 7, where the half-cells were connected by an agarose salt bridge. The cathodic reaction yielded the S-oxidation products of methylphenyl sulfone and methylphenyl sulfoxide, along with *p*-(methylthio) phenol, indicating that the reactive “Cu–O” species is very electrophilic and will prefer oxygen donation even to an activated arene versus a hydrogen abstraction reaction. The electrophilicity is likely related to the strongly electron-withdrawing properties of the high-valent polyoxotungstate framework.^62^

**Scheme 7:**
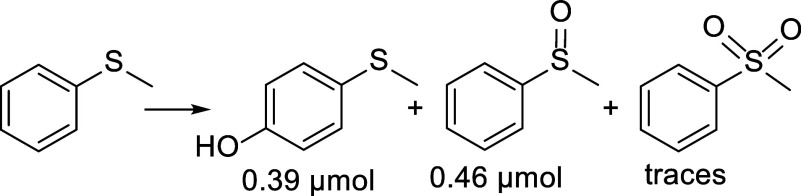
Oxidation of Thioanisole Catalyzed by {Cu_4_(PW_9_)_2_}­^a^

a Cathodic oxidation of thioanisole catalyzed by {Cu_4_(PW_9_)_2_}. Reaction conditions: a divided cell configuration of 10 μmol {Cu_4_(PW_9_)_2_}, 2 μL of thioanisole, and 2.5 mL of D_2_O were placed in each cell. The cells were connected by an agarose salt bridge containing 0.1 M NaClO_4_. Cathode = Pt net. Anode = Pt net. A potential difference of −2 V was applied for 20 h. The products were identified by GC–MS and quantified by ^1^H NMR. The products above were formed in the cathodic compartment. In the anodic compartment, we identified methylphenyl sulfoxide (7.69 μmol), methylphenyl sulfone (0.57 μmol), and small amounts of various disulfides.

In contrast to the oxidation of sulfides, tertiary amines typically undergo dealkylation in the presence of various metal-oxo species through the C–H bond hydroxylation. A thermodynamically controlled reaction will result in the hydroxylation at the α-C–H bond, which has a lower BDEF. On the other hand, a kinetic-oriented reaction will result in hydroxylation at the less hindered β-C–H bond. As can be seen in Scheme 8, the dealkylation of triethylamine can be associated with hydroxylation at the α-C–H bond through the formation of acetaldehyde and acetic acid. The small amount of formic acid formed can be associated with the further oxidation of acetic acid or hydroxylation at the β-C–H bond. This result is different from the one observed using the iron tungstate catalyst {Fe_30_W_72_}, where mostly hydroxylation at the β-C–H position was inferred.^26^

**Scheme 8:**
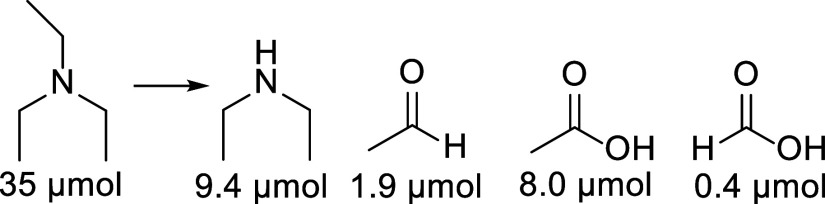
Oxidation of Triethylamine Catalyzed by {Cu_4_(PW_9_)_2_}­^a^

a Reaction conditions: {Cu_4_(PW_9_)_2_} (10 μmol) was dissolved in 2.5 mL of D_2_O inside an airtight electrochemical cell. WE (cathode)—Pt net, surface area 20 cm^2^; CE (anode)—Pt wire, RE—Pt wire; 1 bar of air; 35 μmol of triethylamine; cathodic potential −0.40 V; ∼22 °C; 18 h. CPE was conducted for 18 h. The reaction products were analyzed by ^1^H NMR.

## Conclusions

While the aerobic oxidation of methane to methanol has received much research attention for over 50 years, the oxidation of ethane is much less studied, despite its high concentration in certain natural gas resources, such as those from fracking. Here, we have shown that an inorganic molecular catalyst, {Cu_4_(PW_9_)_2_}, is an efficient catalyst for the cathodic electrocatalytic oxidation of ethane. In an undivided cell, high selectivity to acetic acid can be attained. A turnover frequency (TOF) for {Cu_4_(PW_9_)_2_} can be calculated as follows: TOF = mol_products_/(mol_cat on electrode_ * time), where mol_products_ is the total of all products as shown in Table 1. The surface of the Pt cathode was 20 cm^2^ = 2 *10^15^ nm^2^; the hydrodynamic diameter of {Cu_4_(PW_9_)_2_} was taken as 2.0 nm, which can be translated to a coverage of 3.14 nm^2^ per molecule. At a 1% coverage of {Cu_4_(PW_9_)_2_} on the electrode (see Supporting Information), mol_cat on electrode_ = 20 × 10^15^*0.01/3.14 = 6.4 × 10^13^ molecules = 1.1 × 10^–10^ mol. From this, an intrinsic TOF of about 180 min^–1^ is calculated. This turnover frequency is the same order of magnitude as observed for previous electrocatalytic ethane oxidations.^23,26^

Using a divided cell configuration, it was shown that ethane oxidation to ethanol occurs exclusively at the cathode, while further oxidation to acetaldehyde and acetic acid can occur at both the cathode and anode. Although the interaction between O_2_ and reduced {Cu_4_(PW_9_)_2_} could be inferred through changes in the visible spectrum upon the reaction between {Cu^II^_4_(PW_9_)_2_} and KO_2_, we were not successful in observing any intermediates using various spectroscopic methods under many conditions. We posit the formation of a generic undefined copper-based active species [Cu–O]. Various experiments, including (1) KIE measurements on both aliphatic (1,2 ethanediol) and arene (phenol) substrates and (2) demonstration of hydroxylation at the ipso position of 1,4-difluorobenzene, are inconsistent with hydroxylation by a hydroxyl radical and support a [Cu–O] active species. Further, the use of (3) a dithionite reducing agent (4) labeled ^18^O_2_ and reaction with probe molecules such as (5) 4,4′-cis-stilbene dicarboxylate sodium salt, (6) thioanisole, and (7) triethylamine support a [Cu–O] active species and demonstrate that [Cu–O] likely reacts via a rebound mechanism in the C–H bond hydroxylation of alkanes. On the other hand, the active [Cu–O] species is also very electrophilic as may be deduced from the observed stereo retention in the oxidation of 4,4′-cis-stilbene dicarboxylate sodium salt, the hydroxylation of arenes, and the partial ring hydroxylation of thioanisole. A survey of various distributions of Zn and Cu within the polyoxometalate framework, {Cu_*x*_Zn_4–*x*_(PW)_2_}, in both ensemble and directed positional modes, reveals that at least two and most likely three nearest-neighbor Cu cations are involved in the formation of the active species. Based on a positional analysis of the Cu atoms within {Cu_4_(PW_9_)_2_}, only terminal Cu atoms are good candidates for the initial O_2_ ligation, followed by interaction with the internal Cu atoms. The literature is replete with the isolation of many potential active species upon the reaction of O_2_ with Cu­(I) compounds.^63^ Despite this, the use of organic ligands in this past research and their susceptibility to intramolecular oxidation have limited the use of Cu­(I) complexes for intermolecular oxygenation reactions, notably of alkanes. Therefore, although an actual reactive species for alkane hydroxylation has not been identified, perhaps because of the very high reactivity of [Cu–O], the inorganic polyoxometalate framework is advantageous for intermolecular hydroxylation of alkanes in water. Based on the present results, dinuclear and/or trinuclear bisoxide species involving Cu­(III) may be relevant to explain the reactivity of {Cu_4_(PW_9_)_2_}.^64^

## Experimental Section

### Syntheses of Polyoxometalates

The polyoxometalates, -_4_(H_2_O)_2_(PW_9_O_34_)_2_]^10–^,^27,28^ [Cu_4_(H_2_O)_2_(P_2_W_15_O_56_)_2_]^16–^,^28^ [Cu_3_(PW_9_O_34_)_2_]^12–^,^30^ [Cu_3_(BiW_9_O_34_)_2_]^16–^,^31^ [Cu_2_(H_2_O)_2_SiW_10_O_38_]^8–^,^32^ {NaF_6_Cu­(H_2_O)­W_17_O_55_]^15–^,^33^ and [SiCu_3_(H_2_O)_3_W_9_O_37_]^8–^,^35^ were prepared according the literature procedures.

#### Synthesis of [Na_2_Zn_2_(PW_9_O_34_)_2_]^12–^

According to the literature procedure,^43^ 20 g of Na_2_WO_4_·2H_2_O and 1.21 g of Na_2_HPO_4_·2H_2_O were dissolved in 400 mL of water. 1.24 g of (4.2 mmol) Zn­(NO_3_)_2_·6H_2_O was added to give a cloudy solution. HCl was added dropwise until the pH reached 7.5 and became clear. The solution was then heated to 90 °C for 1 h, cooled to room temperature, and 2 g of KCl was added. The resulting clear solution was left in the fume hood covered with a perforated aluminum foil for crystallization. After 1 month, 2 g (16%) of white crystals were collected and characterized by IR.^43^ IR measurements were carried out on a Nicolet iS50 FTIR instrument.

#### Synthesis of [Na_2_Cu_2_(PW_9_)_2_]^12–^

Adapted from the synthesis of [Na_2_Zn_2_(PW_9_O_34_)_2_]^12–^, 20 g of Na_2_WO_4_·2H_2_O and 1.21 g of Na_2_HPO_4_·2H_2_O were dissolved in 400 mL of water. 0.75 g of CuCl_2_·2H_2_O was added to give a cloudy solution. HCl 37% was added dropwise until the pH reached 7.5, and it became clear green. The solution was then heated to 90 °C for 1 h, cooled to room temperature, and 2 g of KCl was added. The resulting clear green solution was left in the fume hood and covered with a perforated aluminum foil for crystallization. After 1 month, 6 g (47%) of green crystals were collected and characterized by FTIR—1100, 1067, 1038, 1011, 974 (sh), 947, 894, 879, 815, 807, 748 cm^–1^.

#### Synthesis of [Cu_2_Zn_2_(PW_9_)_2_]^10–^

0.1 g of CuCl_2_·2H_2_O (0.588 mmol) was dissolved in 30 mL of 1 M NaCl solution. 1.5 g of [Na_2_Zn_2_(PW_9_O_34_)_2_]^12–^ (0.294 mmol) was added in small portions, and the solution was stirred for 30 min. The slight remaining turbidity was filtered with a filter paper. To precipitate the [Cu_2_Zn_2_(PW_9_)_2_]^10–^, 1.5 g of NaCl was added, followed by 0.75 g of KCl. The solids were collected after 10 min of stirring. The resulting green precipitate was collected and dried under a vacuum. The yield was ∼1.2 g (10%). IR—1039, 1012 (sh), 997, 966, 946, 905, 887, 767, 725 cm^–1^. See the Supporting Information, Table S2.

#### Synthesis of a Mixture of [Cu_*x*_Zn_4–*x*_(PW_9_)_2_]^10–^ *x* = 0–4

CuCl_2_·2H_2_O (0.75 g, 4.5 mmol) and 1.33 g (4.5 mmol) of Zn­(NO_3_)_2_·6H_2_O were dissolved in 30 mL of distilled water. The solid powdered Na_8_H­[A-PW_9_O_34_]·19H_2_0 ^65^ (12.5 g, 4.50 mmol) was added in one portion. The slightly turbid solution was filtered. KCl (1.05 g, 14.1 mmol) was added, and the solution was left to stand as a green precipitate formed. The precipitate was collected and dried under a vacuum. Yield—8.5 g (69%).

### Electrochemical Oxidation Reactions

Electrochemical experiments were carried out using a Biologic multichannel VSP 201 potentiostat. Typically, the required amounts of the catalyst, for example, {Cu_4_(PW)_2_}, were dissolved in D_2_O and placed in an undivided electrolysis cell. The required amount of substrate (gas or liquid) was added while stirring. The electrocatalysis was performed for the given time period by applying a Δ*E* = 2.0 V between the cathode and anode or −0.45 V versus a Pt wire RE. The WE (cathode) was an 80-mesh platinum gauze, and the CE (anode) was a platinum wire. Reactions carried out in D_2_O solution were typically analyzed by ^1^H NMR and ^13^C NMR (11.7 T Bruker ADVANCE III HD Spectrometer). Products were quantified by ^1^H NMR after the addition of dimethylsulfone as a standard by recording 100 scans at the appropriate time intervals. Gas phase analyses of the possible formation of CO and CO_2_ were carried out using an Agilent 6890 gas chromatograph, with a thermal conductivity detector and a ShinCarbon ST 80/100 column from Restek; length, 2.5 m; ID 0.53 μm; He as a gas carrier. The specific conditions for oxidation of all reactions are noted in the table and scheme captions.

### Ethane Oxidation in a Divided Cell Configuration

Experiments were carried out in a 2-electrode setup, where the anodic and cathodic reactions were separated into two different compartments. Each of the electrodes resided in its own vessel, containing a solution of 61.2 mg of polyoxometalate (12 μmol) in 3 mL of D_2_O. The vessels were connected by a U-shaped glass tube that served as a salt bridge. The bridge was 7.5 cm long with an internal diameter of 0.45 cm and a volume of 1.2 mL. It was filled with agarose gel, containing 1 M electrolyte—typically NaCl. The gel was prepared by dissolving 5% agarose (by weight) in a 1 M electrolyte solution and heating it while stirring until the solution became clear. The solution was then decanted into the glass tubes, and it solidified when cooled down. The salt bridges were stored in a 1 M electrolyte solution to prevent them from drying. A Pt net was used as WE and a Pt wire as CE. The entire setup was placed in an airtight electrochemical cell that was pressurized with 1 bar of air and 2 bar of ethane. A potential difference of 2 V was applied between the electrodes.

### Diffusion of Reaction Products between the Anodic and Cathodic Compartments

To have an idea regarding the rate of diffusion between the anodic and cathodic compartments, a solution of methanol, ethanol, and acetic acid was prepared (1 μL each in 3 mL of D_2_O) and was connected to a vessel containing 3 mL of D_2_O either by a salt bridge or by a glass tube containing a Nafion membrane (H-cell). After 18 h, which is a typical reaction time, the concentration of materials on both sides of the H-cell was equal (as determined by ^1^H NMR), meaning that small organic molecules can diffuse easily through the Nafion membrane. Meanwhile, no acetic acid was found to diffuse through the salt bridge in that time frame, and only small amounts of methanol and ethanol (about 20–24%) were found in the other compartment.

### Kinetic Isotope Effect Experiments Ethylene Glycol

163.2 mg (32 μmol) of {Cu_4_(PW)_2_} was dissolved in 8 mL of 90% H_2_O and 10% D_2_O. Protonated and deuterated ethylene glycol (10 μmol each) were added to the solution. The cell was equipped with a magnetic stirring bar, and CPE was performed under an ambient atmosphere using a 2-electrode electrochemical setup. A Pt net was used as the working electrode and a Pt wire as the counter electrode. A potential difference of 2 V was applied between the electrodes for 7 h. During the reaction, samples of 0.5 mL were taken hourly. The protonated reactants and products were analyzed by ^1^H NMR, and the deuterated reactants and products were analyzed by ^2^H NMR. Phenol: 163.2 mg (32 μmol) of {Cu_4_(PW)_2_} was dissolved in 8 mL of D_2_O. Phenol and phenol-*d*_5_ (9 μmol each) were added to the solution. The cell was equipped with a magnetic stirring bar, and CPE was performed under an ambient atmosphere using a 2-electrode electrochemical setup. A Pt net was used as the WE and a Pt wire as the CE. A potential difference of 2 V was applied between the electrodes for 9 h. During the reaction, 9 samples of 0.25 mL each were taken at times of 0.5, 1, 2, 3, 4, 5, 6, 7, and 9 h. For each sample, the amounts of protonated products and the amounts of deuterated products were analyzed by GC–MS.

## Supplementary Material



## Notes

Polyoxometalate structures, IR spectra, ^31^P NMR spectra, CVs, additional CPE experiments, calculations of surface area and coverage, and ICP–OES analyses (PDF)

## Abbreviations

BDEF: bond dissociation free energy
CE: counter electrode
CPE: controlled potential electrolysis
CV: cyclic voltammetry
GC-FID: gas chromatography with a flame ionization detector
GC–MS: gas chromatography with a mass selective detector
ICP–OES: inductively coupled plasma optical emission spectroscopy
IR: infrared
KIE: kinetic isotope effect
NMR: nuclear magnetic resonance
pMMO: particulate methane monooxygenase
RE: reference electrode
TOF: turnover frequency
WE: working electrode
