# Supplementary material for: Copper-Substituted Polyoxotungstates as Catalysts for the Electrocatalytic Oxygenation of Light Alkanes
Source: Inorg Chem. 2025 Sep 4;64(36):18365–75. doi: 10.1021/acs.inorgchem.5c02816 (PMC12442083; doi:10.1021/acs.inorgchem.5c02816)
Supplement: Supplementary file 1 [file ic5c02816_si_001.pdf]

Copper Substituted Polyoxotungstates as Catalysts for the Electrocatalytic Oxygenation of Light Alkanes

Yehonatan Kaufman and Ronny Neumann*

Department of Molecular Chemistry and Materials Science, Weizmann Institute of Science, Rehovot, Israel 7610001

Supporting Information

1) Electrochemical setup:

CPE experiments were conducted either with a 3-electrodes configuration or a 2-electrodes configuration. In a 3-electrodes configuration, Pt net was used as working electrode, Pt wire as counter electrode and another Pt wire was used as a quasi-reference electrode. A fixed potential was set between the working electrode and the reference electrode. In a 2-electrodes configuration, the quasi-reference electrode was omitted. A fixed potential was set between the working and counter electrodes ("cell voltage").

Note that cyclic voltammetry was measured using an Ag/AgCl reference electrode. As is known, this electrode is composed of a silver wire placed inside a tube containing KCl solution, and therefore not compatible with our pressure electrochemical reactor since the pressure of gaseous substrate will push the KCl solution out of the electrode. See Figure S1 for our electrochemical setups.

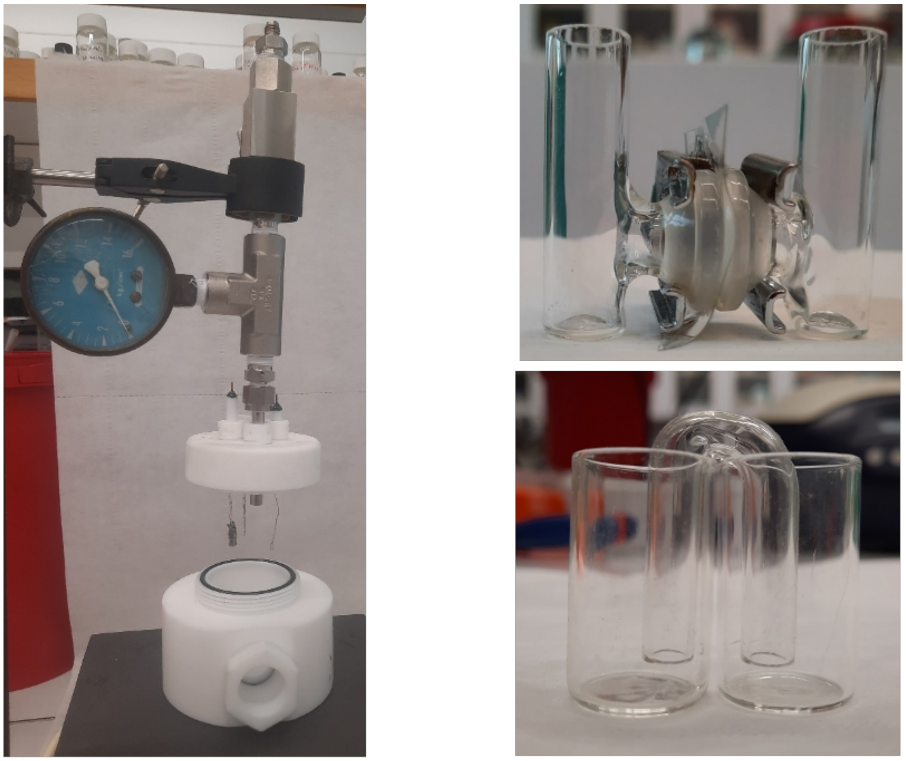

Figure S1: electrochemical setup. Left – pressure electrochemical reactor, fitted with a Pt gauze working electrode, Pt wire counter electrode and Pt wire quasi-reference electrode. Top right – H-cell configuration, where the two glass vessels are separated by a nafion membrane. Bottom right – salt bridge configuration, where the two glass vessels are connected by a glass tube containing agarose gel and electrolyte. Both the H-cell and the salt bridge can fit inside our pressure electrochemical reactor.

2) Determining the required potential for CPE:

To determine the required potential for a CPE experiment, a cyclic voltammetry was measured under the reaction conditions using a Pt wire as quasi-reference electrode. The chosen potential was -0.4V vs Pt, which corresponds to about -0.15 V vs Ag/AgCl, see Figure S2.

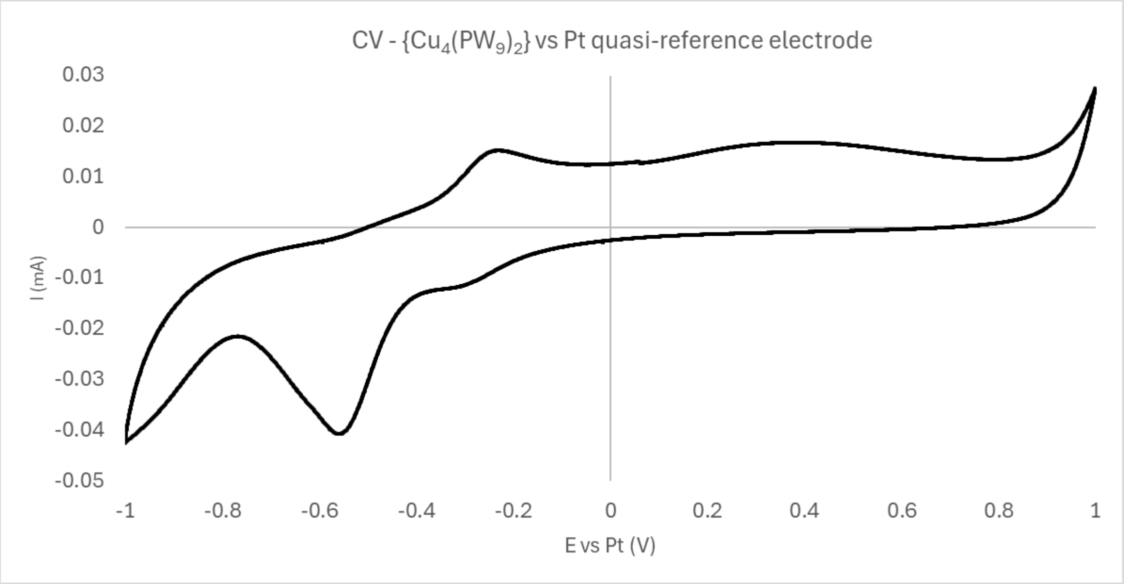

Figure S2: cyclic voltammogram of $\{Cu_4(PW_9)_2\}$ under the reaction conditions. A solution of 4mM POM and no supporting electrolyte was measured using Pt disk working electrode, Pt wire counter electrode and Pt wire quasi-reference electrode. A potential of -0.45 V was chosen for the CPE experiments, as it after the onset potential of the surmised Cu^{II}-$O_2^{-\bullet}$ specie, but before its peak to prevent Cu^0 deposition.

3) Switching from 3-electrodes configuration to 2-electrodes configuration:

When performing a CPE experiment with 3-electrodes configuration, the cell potential, which is the ΔV between the working electrode and the counter electrode, was measured. This potential was found to be -2V. This value was used when performing CPE experiments with 2-electrodes configuration.
In addition, a series of CPE experiments at different cell potentials were conducted. We found that a ΔV of -2V gave the best results in terms of faradic efficiency and the amount of products, See Table S1.

ΔV	Ethanol (umol)	Acetaldehyde (umol)	Acetic acid (umol)	Faradic efficiency
-1.7	0.32	0.81	1.41	14%
-1.9	0.47	1.7	3.54	22%
-2	0.63	3.34	9.37	39%
-2.1	0.55	1.36	1.86	12%
-2.2	0.35	0.73	4.43	8%

Table S1: A series of CPE experiments at different cell potential. Conditions: 51 mg $\{Cu_4(PW_9)_2\}$ (10 umol) were dissolved in 2.5 ml D_2O and placed under pressure of 1 atm air and 2 atm ethane. A CPE was performed for 18h using 2-electrodes configuration and different cell potentials, ranging from -1.7V to 2.2V. product analysis was done using H-NMR.

4) Recycling and reusing the catalyst:

After a CPE reaction (cell voltage – 2 V or cathodic potential -0.45 V, no or only traces of Cu^0 was deposited on the cathode. This was measured by dissolution of any residual Cu^0 by 1 M nitric acid and measurement of the visible spectrum versus a calibration curve of $Cu(NO_3)_2$. In addition, after a CPE reaction, an excess of KCl was added to the reaction solution (50 mg KCl for every 1 ml solution) causing the $\{Cu_4(PW_9)_2\}$ catalyst to immediately precipitate. The precipitation was collected by centrifugation and dried at high vacuum for 18h. The IR spectrum of the precipitation was identical to the spectrum of freshly prepared material. Using the recycled polyoxometalate in a CPE experiment gave the same results compared to a fresh catalyst.

5) Determining the surface area of the Pt net cathode:

The surface area of the Pt-gauze cathode was determined by electrochemical absorption/desorption of H^+ in acidic solution, as was publish by Yang, Shao-Horn at al.[1] In short, a cyclic voltammetry was conducted using a Pt-gauze working electrode, Pt wire counter electrode and Ag/AgCl reference electrode. The analyte was a 0.5M H_2SO_4 solution saturated with argon. Initially, a potential window of 1.15 V to -0.175 V was measured. The voltammogram was measured several times, each time increasing the potential window toward larger negative values, until the onset potential of H^+ reduction was observed at -0.185V, see Figure S4. The anodic peaks (yellow at Figure S4) correspond to the oxidative desorption of adsorbed H^+ species:

$$H^* \rightarrow H^+ + e^-$$

The area of the peak equals the charge that passed from the electrode to the adsorbed species. Multiplying the charge by the experimental constant of polycrystalline platinum (176 µC/cm^2) gives a surface area of about 20 cm^2.

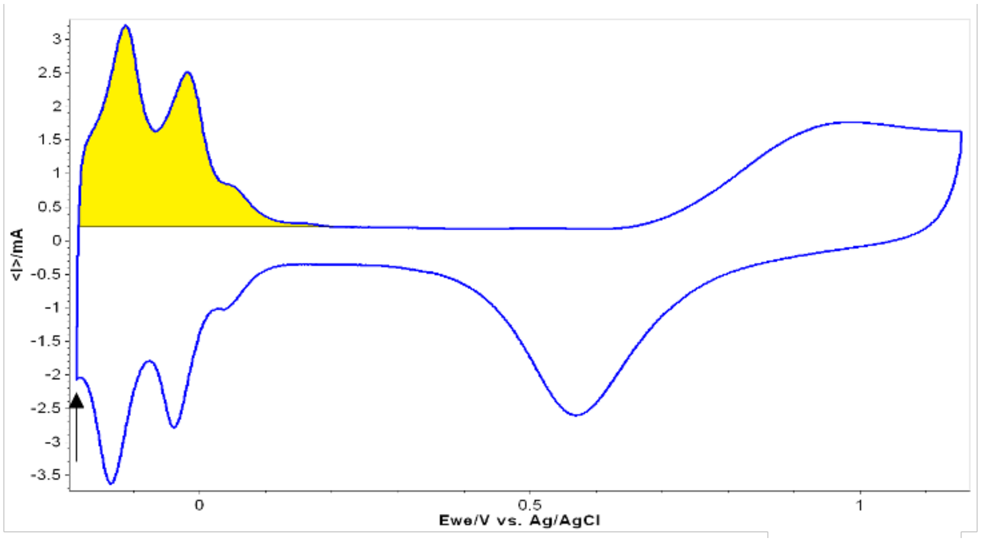

Figure S3: Calculating the surface area of a Pt-gauze working electrode. Conditions: solution of 0.5 M H_2SO_4 saturated with Ar. Working electrode – Pt-gauze, counter electrode – Pt wire, reference electrode – Ag/AgCl. The black arrow indicates the onset potential of H^+ reduction, which defines the left boundary of the scan. The yellow area corresponds to the oxidative desorption of *H species and correlated to the surface area of the electrode.

6) Additional cyclic voltammograms:

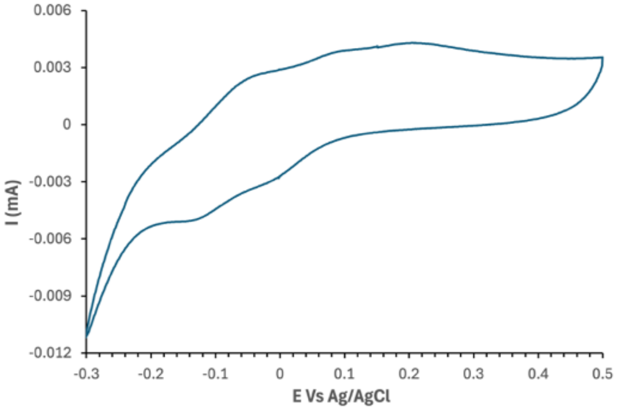

Figure S4: Cyclic voltammetry of $\{Cu_4(PW_9)_2\}$ in 0.1 M $NaClO_4$ under 1 bar Ar. Working electrode (WE) – glassy carbon disc, counter electrode (CE) - Pt wire, reference electrode (RE) – Ag/AgCl.

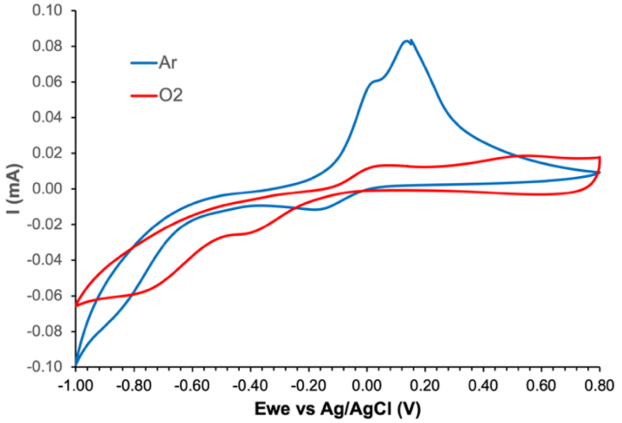

Figure S5. Cyclic voltammetry of $\{SiCu_3(H_2O)_3W_9O_{34}\}$ in 0.1 M $NaClO_4$ under 1 bar Ar or O_2. Working electrode (WE) – glassy carbon disc, counter electrode (CE) - Pt wire, reference electrode (RE) – Ag/AgCl.

7) structure of the different copper-polyoxometalate used in this work:

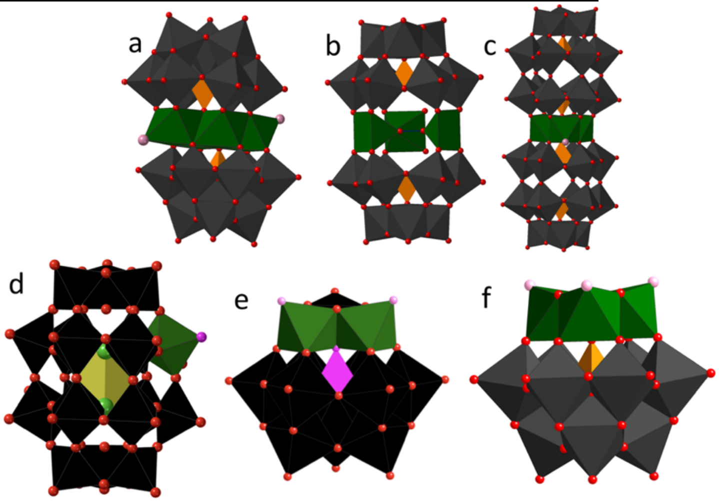

Figure S6. Polyhedral Structures of Different Cu Substituted Polyoxometalates. Black - W; Red – O; Dark Green – Cu; Orange– P, Si or Bi; Magenta-Si; Yellow-green-Na; Light green-F; Pink – H_2O. a - $\{Cu_4(PW_9)_2\}$; b - $\{Cu_3(PW_9)_2\}$ and $\{Cu_3(BiW_9)_2\}$ (terminal H_2O and NO_3^- not shown); c - $\{Cu_4(P_2W_{15}O_{56})_2\}$; d - $\{CuNaF_6W_{17}\}$; e - $\{Cu_2PW_{10}\}$; f - $\{SiCu_3W_9\}$.

8) Aerobic oxidation of additional substrates catalyzed by {$Cu_4(PW_9)_2$}:

Scheme S1. Cathodic Oxidation of Propane Catalyzed by {$Cu_4(PW_9)_2$}

$CH_3CH_2CH_3$ ⟶	$CH_3CHOHCH_3$ +	$CH_3C(O)CH_3$ +	CH_3CH_2COOH +	CH_3CHO +	CH_3COOH +	HCHO +	HCOOH
FE - 42%	0.05 *μ*mol	12.8 *μ*mol	3.0 *μ*mol	0.76 *μ*mol	10.6 *μ*mol	0.16 *μ*mol	1.1 *μ*mol

Reaction conditions: {$Cu_4(PW_9)_2$} (10 μmol) was dissolved in 2.5 mL D_2O inside airtight electrochemical cell. A 3 electrodes configuration was used. Working electrode (cathode)- Pt net, surface area 20 cm^2; counter electrode (anode)-Pt wire, RE -Pt wire.; 1 bar air; 2 bar propane; cathodic potential -0.40 V (-0.15 V vs SCE); ~22 °C; 24 h. Liquid phase product analysis by ^1H NMR using dimethyl sulfone as internal standard introduced post-reaction.

Scheme S2. Cathodic Oxidation of Ethylene Catalyzed by {$Cu_4(PW_9)_2$}.

CH_2=CH_2 ⟶	$HOCH_2CH_2OH$ +	$OHCCH_2OH$ +	HCHO +	HCOOH
FE - 36%	7.0 *μ*mol	2.0 *μ*mol	6.7 *μ*mol	9.0 *μ*mol

Reaction conditions: {$Cu_4(PW_9)_2$} (10 μmol) was dissolved in 2.5 mL D_2O inside airtight electrochemical cell. A 3 electrodes configuration was used. Working electrode (cathode)- Pt net, surface area 20 cm^2; counter electrode (anode)-Pt wire, RE -Pt wire.; 1 bar air; 2 bar ethylene; cathodic potential -0.40 V (-0.15 V vs SCE); ~22 °C; 24 h. Liquid phase product analysis by ^1H NMR using dimethyl sulfone as internal standard introduced post-reaction.

Scheme S3. Cathodic Oxidation of Propylene Catalyzed by {$Cu_4(PW_9)_2$}.

CH_2=$CHCH_3$ ⟶	$HOCH_2CHOHCH_3$ +	CH_3CHO +	CH_3COOH +	HCHO +	HCOOH
FE - 31%	1.8 *μ*mol	2.8 *μ*mol	4.2 *μ*mol	3.4 *μ*mol	4.4 *μ*mol

Reaction conditions: {$Cu_4(PW_9)_2$} (10 μmol) was dissolved in 2.5 mL D_2O inside airtight electrochemical cell. A 3 electrodes configuration was used. Working electrode (cathode)- Pt net, surface area 20 cm^2; counter electrode (anode)-Pt wire, RE -Pt wire.; 1 bar air; 2 bar propylene; cathodic potential -0.40 V (-0.15 V vs SCE); ~22 °C; 24 h. Liquid phase product analysis by ^1H NMR using dimethyl sulfone as internal standard introduced post-reaction.

9) ^{31}P NMR spectra of mixed-belt polyoxometalates

As we mentioned in the manuscript, the mixed-belt polyoxometalate of {$Cu_xZn_{4-x}(PW_9)_2$} was prepared by reacting a 1:1 mixture of Cu^{2+} and Zn^{2+} with $[PW_9O_{34}]^{9-}$. We posit that the product is an ensemble of several sandwich-type polyoxometalates, each with different ratio of copper and zinc. To verify this assumption, we used the analogous sandwich-type polyoxometalate of {$M_4(P_2W_{15}O_{56})$}. This "elongated" version of {$M_4(PW_9O_{34})_2$} has an identical belt structure and larger tungsten-oxide frameworks. This polyoxometalate has 4 phosphorus atoms – 2 of them are proximal to the belt and the other 2 are distal to the belt. The 2 distal phosphorus atoms are visible by ^{31}P NMR even when paramagnetic atoms, such as copper are present. In contrast, the 2 phosphorus atoms of {$M_4(PW_9O_{34})_2$} are not visible in P-NMR when paramagnetic atoms are present. See Figure S7 for the structure of {$M_4(PW_9O_{34})_2$} and {$M_4(P_2W_{15}O_{56})$}. As can be seen in Figure S11, the polyoxometalate of {$Zn_4(P_2W_{15}O_{56})$} has two peaks in its P-NMR spectrum – one for the proximal phosphorus (-4.5 ppm) and one for the distal phosphorus (-14.5 ppm). The polyoxometalate of {$Cu_4(P_2W_{15}O_{56})$} has only one broad phosphorus peak at -17 ppm, corresponding to the distal phosphorus atoms. The {$Cu_xZn_{4-x}(P_2W_{15}O_{56})$} polyoxometalate was synthesized in the same manner as {$Cu_xZn_{4-x}(PW_9)_2$}, but with $[P_2W_{15}O_{56}]^{12-}$ instead of $[PW_9O_{34}]^{9-}$. The ^{31}P NMR spectrum contains the two peaks of {$Zn_4(P_2W_{15}O_{56})$}, the single peak of {$Cu_4(P_2W_{15}O_{56})$}, and many other peaks – associated with mixed-Cu, Zn polyoxometalates with different ratio of copper and zinc.

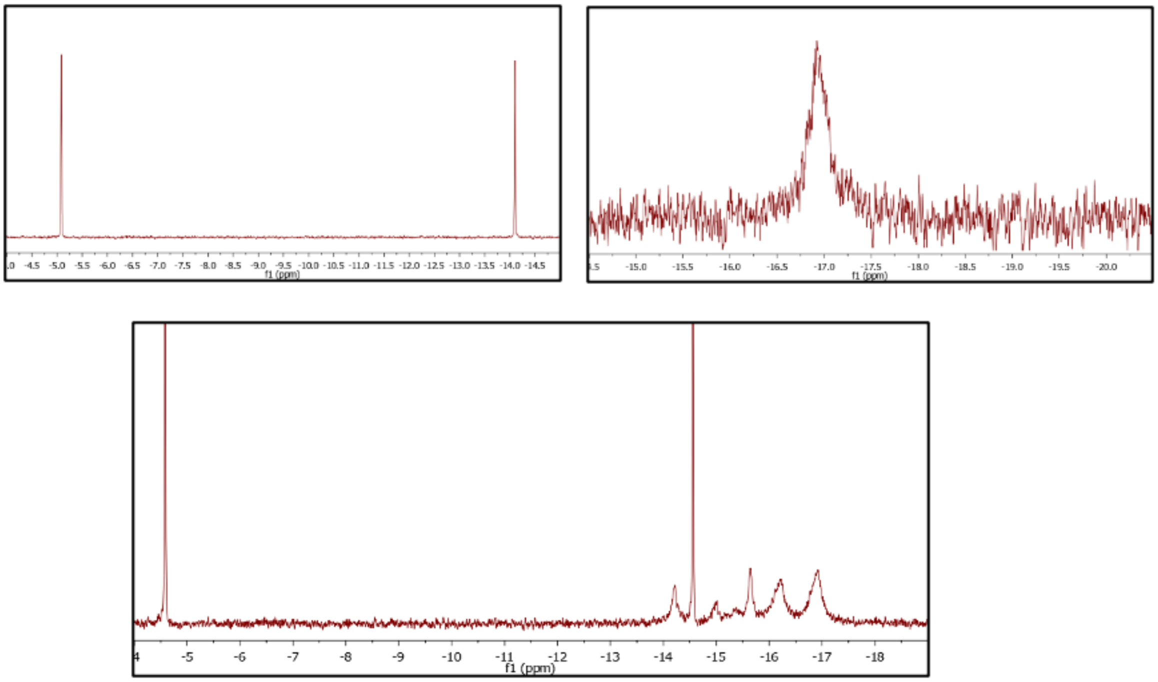

Figure S7: P-NMR spectra of sandwich-type polyoxometalates. Top left: $\{Zn_4(P_2W_{15}O_{56})\}$ has two sharp P-NMR peaks, one for the proximal P atoms and one for the distal P atoms. Top right: $\{Cu_4(P_2W_{15}O_{56})\}$ has only one broad P-NMR peak, representing the distal P atoms. bottom: The mixed-belt $\{Cu_xZn_{4-x}(P_2W_{15}O_{56})\}$ has several ^{31}P NMR peaks, each representing a different copper, zinc composition.

10) IR spectra of mixed belt polyoxometalates

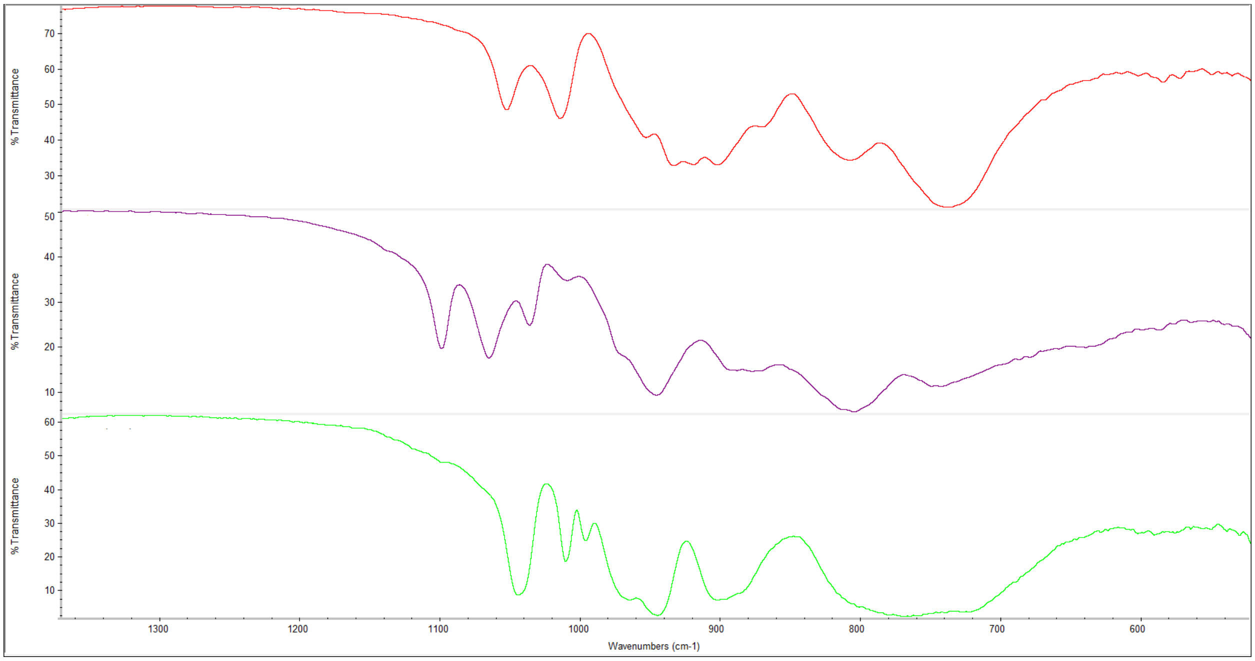

Figure S8. IR Spectra of $\{Na_2Zn_2(PW_9)_2\}$ (top), $\{Zn_2Cu_2(PW_9)_2$ (middle), and $\{Cu_4(PW_9)_2\}$.

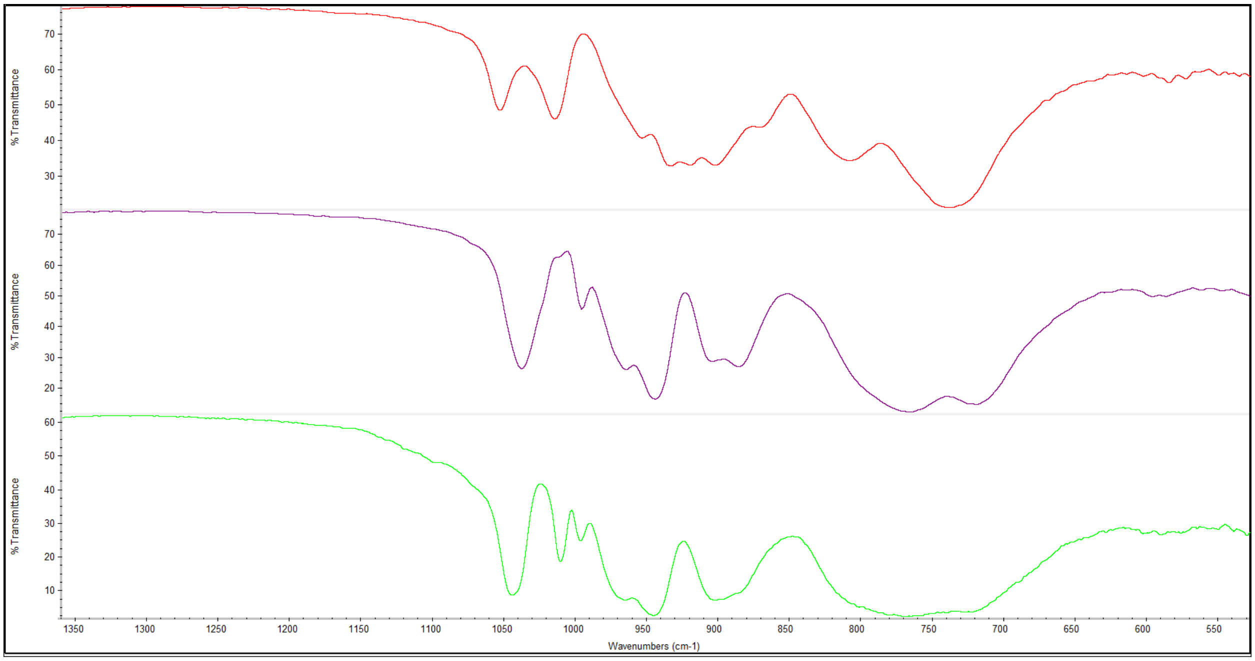

Figure S9. IR Spectra of $\{Na_2Zn_2(PW_9)_2\}$ (top), $\{Cu_2Zn_2(PW_9)_2$ (middle), and $\{Cu_4(PW_9)_2\}$.

<u>11) Cyclic voltammogram of mixed metal polyoxometalates</u>

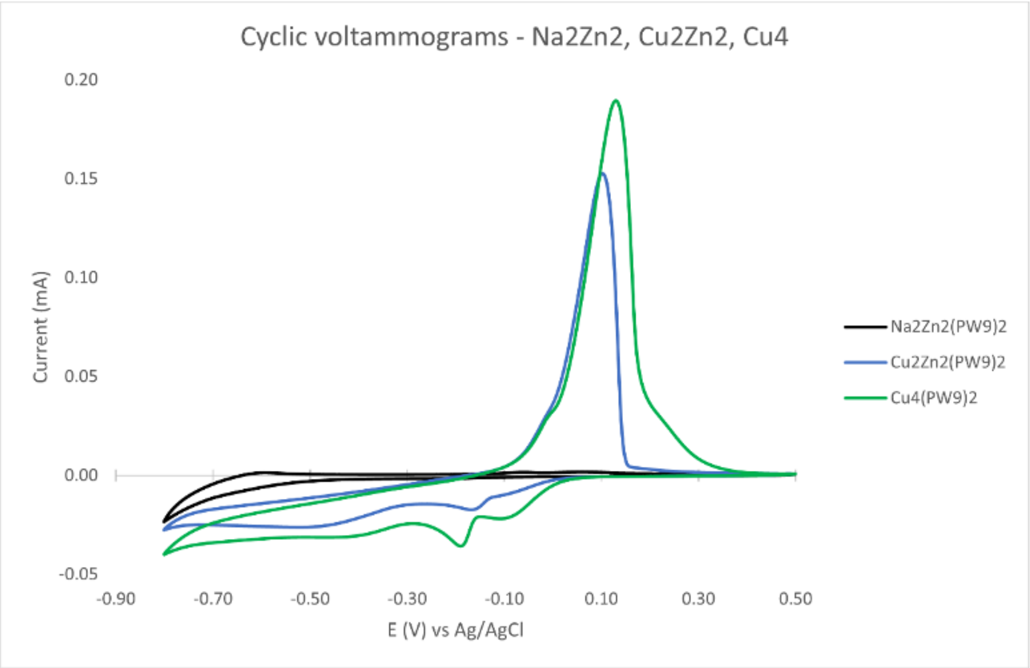

Figure S10: Cyclic voltammograms of $\{Na_2Zn_2(PW_9)_2\}$ (black), $\{Cu_2Zn_2(PW_9)_2\}$ (blue) and $\{Cu_4(PW_9)_2\}$ (green). Conditions: aqueous solution containing 4mM POM and 0.1M $NaClO_4$ as supporting electrolyte. Working electrode – glassy carbon disk, counter electrode – Pt wire, reference electrode – Ag/AgCl.

12) Elemental analysis of mixed belt polyoxometalates:

The compositions of the mixed-belt polyoxometalates were measured by using ICP-OES. The results are presented at Figure S15, normalized by the expected number of tungsten atoms in each compound.

Polyoxometalate	W	P	Cu	Zn
$\{Cu_4(PW_9)_2\}$	18	2.18	4.85	0
$\{Na_2Zn_2(PW_9)_2\}$	18	2.03	0	2.24
$\{Na_2Cu_2(PW_9)_2\}$	18	2.04	2.53	0
$\{Cu_2Zn_2(PW_9)_2\}$	18	2.17	2.22	1.97

Table S2: ICP-OES measurement of $\{Cu_4(PW_9)_2\}$ and 3 different mixed-belt polyoxometalates.

13) Competitive kinetic isotope effect experiments:

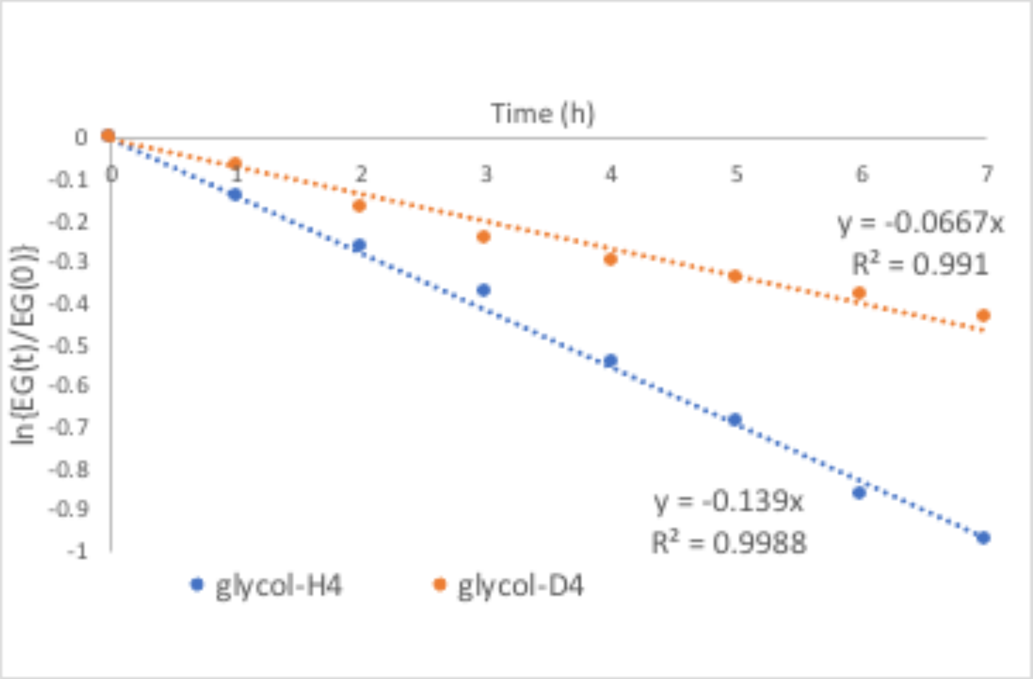

Figure S11. Competitive KIE experiment using ethylene glycol as substrate. Conditions: 32 umol $\{Cu_4(PW_9)_2\}$ were dissolved in 8 ml solution of 90% H_2O and 10% D_2O. Protonated and deuterated ethylene glycol (10 umol each) were added. WE=Pt net, CE=Pt wire. A CPE was performed for 8h by applying a potential difference of -2V between the electrodes. Samples of 0.5 mL were extracted every hour, and the consumption of both substrates were analyzed via ^1H NMR and ^2H NMR.

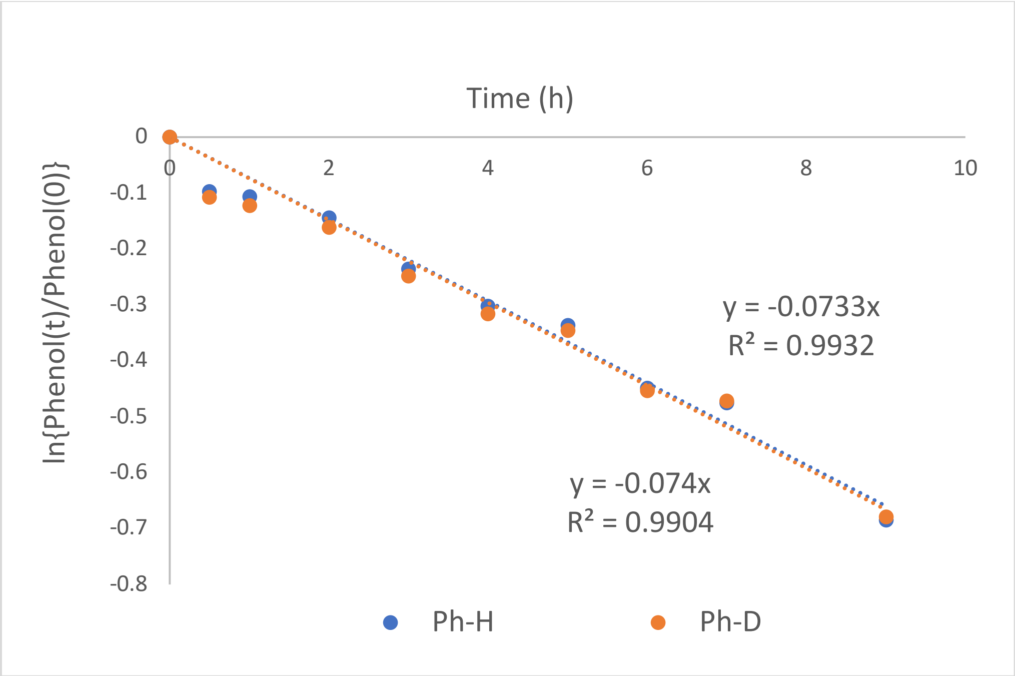

Figure S12. Competitive KIE experiment using phenol as substrate. Conditions: 32 umol $\{Cu_4(PW_9)_2\}$ were dissolved in 8 mL D_2O. Protonated and deuterated phenol (about 9 umol each) were added. Two electrodes setup was used. WE=Pt net, CE=Pt wire. A CPE was performed for 9h by applying a potential difference of -2V between the electrodes. During the electrolysis, 9 samples of 0.25 ml were extracted. The consumption of protonated substrate was analyzed via ^{1}H NMR, and the consumption of deuterated substrate was analyzed via GC-MS.

14) Coverage of the $\{Cu_4(PW_9)_2\}$ on the electrode

When calculating the turnover frequency (TOF), we assume that 1% of the cathode's surface is covered by $\{Cu_4(PW_9)_2\}$ at any given time. We derived this value by assuming that due to the constant stirring, the catalyst is uniformly dispersed in the solution. the coverage of the electrode is therefore calculated by:

$$coverage = \frac{(concentration\ of\ catalyst) * (volume\ of\ a\ catalyst\ molecule)}{(concentration\ of\ solvent) * (volume\ of\ a\ solvent\ molecule)}$$

The concentration of the catalyst is 4 mM and the concentration of solvent (water) is 55.55M. The volume of a catalyst and solvent molecules can be derived from their hydrodynamic radius, which is 1 nm and 0.2nm respectively. Overall, the calculated value was 0.9%, so using a coverage of 1% in our calculation is a reasonable estimation.

Reference

Wei, R.; Peng, H.; Stephens, R.; Xu, S.-H. Recommended Practices and Benchmark Activity for Hydrogen and Oxygen Electrocatalysis in Water Splitting and Fuel Cells"; *Adv. Mater.* **2019**, 1806296.
